# Translational development of a tumor junction opening technology

**DOI:** 10.1038/s41598-022-11843-z

**Published:** 2022-05-11

**Authors:** Jiho Kim, Chang Li, Hongjie Wang, Swarnendu Kaviraj, Sanjay Singh, Laxman Savergave, Arjun Raghuwanshi, Sucheol Gil, Audrey Germond, Audrey Baldessari, Bingmae Chen, Steve Roffler, Pascal Fender, Charles Drescher, Darrick Carter, André Lieber

**Affiliations:** 1 Division of Medical Genetics, School of Medicine, University of Washington, Seattle, WA USA; 2 PAI Life Sciences, Seattle, WA USA; 3 Gennova Biopharmaceuticals, Ltd, Pune, India; 4 Washington National Primate Research Center, Seattle, WA USA; 5 Institute of Biomedical Sciences, Academia Sinica, Taipei, Taiwan; 6 CNRS, Univ. Grenoble Alpes, CEA, UMR5075, Institut de Biologie Structurale, 38042 Grenoble, France; 7 Public Health Sciences Division, Fred Hutchinson Cancer Research Center, Seattle, WA USA; 8 Department of Global Health, School of Public Health, University of Washington, Seattle, WA USA; 9 Department of Pathology, School of Medicine, University of Washington, Box 357720, Seattle, WA 98195 USA

**Keywords:** Cancer microenvironment, Cancer therapy, Gynaecological cancer

## Abstract

Our goal is to overcome treatment resistance in ovarian cancer patients which occurs in most cases after an initial positive response to chemotherapy. A central resistance mechanism is the maintenance of desmoglein-2 (DSG2) positive tight junctions between malignant cells that prevents drug penetration into the tumor. We have generated JO4, a recombinant protein that binds to DSG2 resulting in the transient opening of junctions in epithelial tumors. Here we present studies toward the clinical translation of c-JO4 in combination with PEGylated liposomal doxorubicin/Doxil for ovarian cancer therapy. A manufacturing process for cGMP compliant production of JO4 was developed resulting in c-JO4. GLP toxicology studies using material from this process in DSG2 transgenic mice and cynomolgus macaques showed no treatment-related toxicities after intravenous injection at doses reaching 24 mg/kg. Multiple cycles of intravenous c-JO4 plus Doxil (four cycles, 4 weeks apart, simulating the treatment regimen in the clinical trial) elicited antibodies against c-JO4 that increased with each cycle and were accompanied by elevation of pro-inflammatory cytokines IL-6 and TNFα. Pretreatment with steroids and cyclophosphamide reduced anti-c-JO4 antibody response and blunted cytokine release. Our data indicate acceptable safety of our new treatment approach if immune reactions are monitored and counteracted with appropriate immune suppression.

## Introduction

Epithelial junctions that link cancer cells create physical barriers to the intratumoral penetration of therapeutic agents. Most drugs—and in particular nanoparticle- or liposome-based drugs (diameters ~ / > 100 nm)—do not diffuse more than a few cell layers from blood vessels implying that more distant tumor cells receive only sub-therapeutic drug exposure^1–3^. A major junction protein associated with this resistance is desmoglein-2 (DSG2). We have previously reported that the degree of DSG2 expression correlated with ovarian cancer grade and treatment resistance^4^.

DSG2 is also used as a receptor by species B human adenoviruses^5^. Among DSG2-targeting viruses is serotype 3 (Ad3). Ad3 is able to efficiently breach the epithelial barrier in the airway tract and infect airway epithelial cells. We found that this is achieved by the binding of an Ad3 fiber capsid protein to DSG2 and subsequent intracellular signaling, that results in transient opening of tight junctions between epithelial cells^6^. We have capitalized on this mechanism and created recombinant proteins that contain the minimal structural domains from Ad3 that are required for junction opening^7,8^. We have shown that intravenous injection of junction opener proteins increases intratumoral penetration and efficacy of monoclonal antibodies and chemotherapeutic drugs in a broad range of human xenograft models^9,10^.

JO4 is a rationally designed protein with an artificial dimerization domain and specific mutations that greatly increase the affinity to DSG2 in a way that triggers junction opening more efficiently than the parental Ad3 virus^11^. Disruption of junctions by JO4 is transient and the junction structure is completely restored in a short time period after JO4 is eliminated. JO4 action is tumor-specific because DSG2 in normally polarized epithelial tissues is trapped in lateral junctions and not accessible to JO4.

We plan to test JO4 in combination with PEGylated liposomal doxorubicin (PLD) clinically in ovarian cancer patients. PLD is marketed under the names Doxil, Caelyx, or Lipodox. The liposomal formulation is formed of a polyethylene glycol (PEG) coat on the exterior protruding from an amphipathic bilayer. The core is comprised of a single nanocrystal aqueous in the liposome core. The size of the liposome is approximately 100 nm, which precludes it from capillary junctions such as those found in the heart, further decreasing the possibility of cardiotoxicity and fatal side effects^12^. As such, Doxil has been approved for use in platinum-resistant ovarian cancer^13^. However, response rates to Doxil are low, the response duration is short, and the toxicity is significant^14–16^. There is a great need for improvement of both the efficacy and the safety of Doxil therapy in ovarian cancer patients. The goal of our c-JO4 approach is to increase Doxil accumulation in the tumor by opening epithelial junctions, thereby decrease the exposure of normal tissue to Doxil and reduced side effects of this drug.

Here we performed toxicology studies for JO4 + Doxil combination therapy as part of the preparation of an Investigational New Drug (IND) submission. The studies were performed with JO4 drug substance that was manufactured based on a cGMP-compliant protocol and that had clinical-grade quality (c-JO4). We used two adequate animal toxicology models: DSG2 transgenic mice and cynomolgus monkeys. The homology between the human and mouse DSG2 gene is 77.1% and neither Ad3 nor JO4 bind to mouse cells^17^. We therefore generated transgenic mice that contain the 90 kb human DSG2 locus including all regulatory regions. These mice express human DSG2 in a pattern and at a level similar to humans^17^. In the past we accumulated a substantial amount of efficacy and safety data in hDSG2 transgenic mice^6,7,11,17^. JO4 triggers junction opening in hDSG2-expressing epithelial mouse tumor cells indicating that hDSG2 interacts with mouse signaling and cytoskeletal proteins thus overriding a potential function of mouse DSG2 in the maintenance of junctions in transgenic mice. Nonetheless, a DSG2 animal model with a homologous downstream signaling machinery would be preferable. The DSG2 gene homology between humans and macaques is 96.6%. Biodistribution in cynomolgus monkeys (*Macaca fascicularis)* is similar to humans^17^. JO4 binds to monkey DSG2 and triggers junction opening at a level that is comparable to its effect on human cells^17^.

Overall, our studies showed that c-JO4 + Doxil treatment is well tolerated. Besides defining the highest non-severely toxic dose (HNSTD) of c-JO4 in NHPs, we used the NHP model to assess c-JO4 immunogenicity after multiple treatment cycles. The data accumulated will be included as part of the preclinical sections of an IND application.

## Results

### Bridging from Research Material to Industrial cGMP-compliant product

Previously produced JO4 research material (“r-JO4”) had problems with yields, endotoxin, and aggregation and would not be suitable for clinical development^7^. We therefore worked with a cGMP compliant manufacturing organization, Gennova Biopharmaceuticals (Pune, India) which developed a new purification protocol that could be used for cGMP compliant production of c-JO4. The protocol involved affinity resin purification followed by a refolding step to yield partially purified, active c-JO4. The protein was then put through a size exclusion step to remove high molecular weight aggregates and then polished using an ion exchange resin to remove residual endotoxins and other host cell contaminants like proteins and nucleic acids. The final product was then buffer exchanged into PBS, 5% D-Sorbitol, pH 7.4 buffer and sterile filtered to yield the infusible product at a yield of about 0.7 g per liter of fermented bacterial bulk.

**Figure 1:**
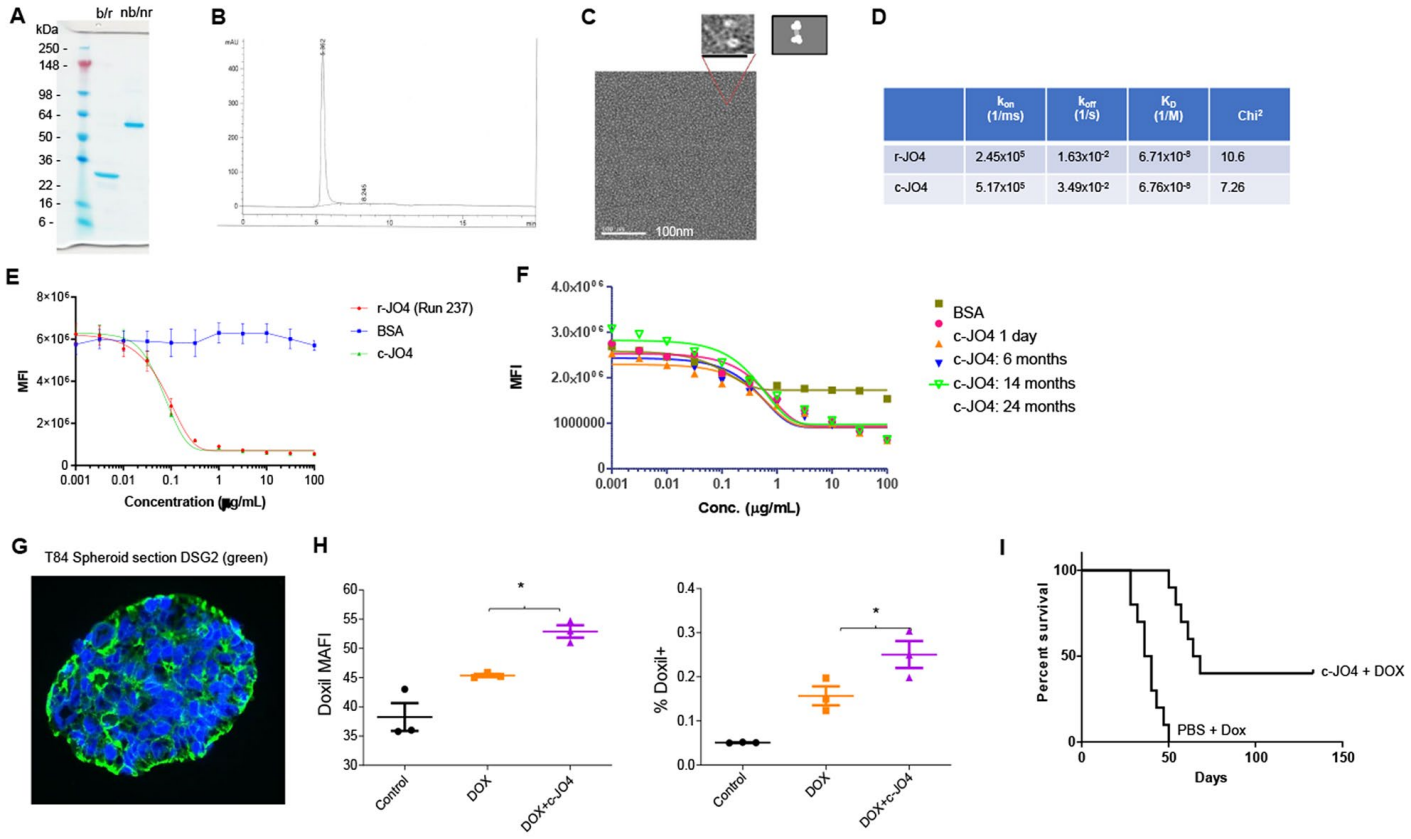
Characterization of c-JO4. (**A**) Polyacrylamide gel electrophoresis. Gennova’s preparation of JO4 (c-JO4) was treated as indicated. b/r—boiled and reduced; nb/nr—not boiled and not reduced. 1 μg protein was run on the gel. (**B**) Size exclusion chromatography (SEC) profile of c-JO4 run through a Superdex 200 increase 10/300 column with TBS. (**C**) Electron microscopy of c-JO4 negative staining with sodium silicotungstate. The zoomed in image showed a dimer of trimeric fiber knob domains. A schematic illustration of this structure is shown on the right. (**D**) Surface Plasmon Resonance analysis of JO4 binding to recombinant DSG2. DSG2 was immobilized on sensorchips, and background was automatically subtracted from the control flow cell. r-JO4 and c-JO4 were injected for 3 min at 2.5 μg/ml, followed by a 2.5-min dissociation period. Shown is the summary of SPR data. Parameters were evaluated using BIAeval software. k_on_- association rate; k_off_—dissociation rate; K_D_—equilibrium dissociation constant; chi^2^—area of the squared difference between the measured data point and the fit. (**E**–**I**) Potency assays. (**E**) Ad3-GFP Infection competition. Research r-JO4 run (#237) was compared with c-JO4. 293 cells were incubated in 96-well plates with JO4 proteins at indicated concentrations for 1 h before addition of Ad3-GFP virus for competition. GFP expression was measured 18 h after virus addition to measure extent of JO4 competition with Ad3-GFP. (**F**) c-JO4 was stored at 4 °C for 1 day (red dot), 6 months (orange triangle), 14 months (blue triangle), and 24 months (empty green triangle) and then tested in infection competition assay. (**G,H**) c-JO4-enhanced Doxil penetration in epithelial tumor spheroids. (**G**) Confocal immunofluorescence images of T84 spheroids stained for DSG2 (Green) and DAPI (blue). (**H**) Left panel: Uptake of liposomal doxorubicin (Doxil) (DOX) in T84 cell spheroids after treatment with c-JO4. The amount of Doxil (mean autofluorescence intensity—MAFI) in tumor cells was measured 1 h after adding it to tumor sphere cultures. Right panel: Doxil released from spheroids into the supernatant after trypsin digestion of spheroids. *p < 0.05. (**I**) c-JO4-enhanced anti-tumor activity. CB17 immunodeficient mice bearing epithelial tumors derived from T84 human colon adenocarcinoma cells were used for testing. When tumors reached a volume of 100 mm^3^, mice were intravenously injected with either PBS or c-JO4 (2 mg/kg) followed by Doxil (1 mg/kg) one hour later. Mice received three injection cycles 3 days apart. Tumor volumes were measured twice a week until reaching a tumor volume of 1000 mm^3^ when they were sacrificed. The day of sacrifice served as the endpoint in Kaplan–Meier survival studies. In the c-JO4/Doxil group, tumor growth was significantly delayed (p < 0.0001) and 45% of the animals were still tumor-free at day 140 after tumor cell inoculation. N = 10. p < 0.001 for Doxil vs c-JO4 + Doxil.

This c-JO4 product had a purity of > 99% with endotoxin levels of < 200 EU/ml. c-JO4 was characterized by biophysical methods (Fig. 1). In agreement with previous r-JO4 data^7,8^, even in the presence of 0.1% SDS, the c-JO4 dimer-trimer migrates in a polyacrylamide gel at about 65 kDa—likely as an assembled trimer (Fig. 1A). Boiling in the presence of a reducing agent resulted in dissociation into monomers with a molecular weight of ~ 25 kDa. The new refolding technology used for manufacturing of c-JO4 resulted in a product with only minimal aggregation. Both size-exclusion chromatography (SEC) (Fig. 1B) and electron microscopy (Fig. 1C) show uniform c-JO4 dimers of trimers of 11 nm in length with a maximum diameter of 7 nm. Surface Plasmon Resonance analysis using immobilized recombinant DSG2 protein showed similar equilibrium dissociation constants (K_D_) for r-JO4 and c-JO4 (Fig. 1D).

We performed a series of potency studies with c-JO4 in comparison to r-JO4 to bridge the large amount of efficacy data that we previously obtained with r-JO4 (Fig. 1E–I). The first assay measured the ability of JO4 to block the infection of 293 cells by a GFP-expressing Ad5/3 adenovirus vector that has the same tropism to DSG2 as JO4 (Fig. 1F). This assay did not show differences between both JO4 preparations. It also showed that c-JO4 did not lose potency after storage at 4 °C for up to two years (last time point analyzed) (Fig. 1E). Furthermore, we used 3D spheroid cultures formed by colon cancer T84 tumor cells to analyze the effect of c-JO4 on Doxil uptake. T84 spheroids form typical epithelial junctions that stain for DSG2 (Fig. 1G). Doxil was added to the spheroid culture medium in the presence or absence of c-JO4. One hour later, the spheroids were washed and digested with trypsin to obtain single cell suspension. The amount of Doxil (based on its mean autofluorescence intensity—MAFI) inside cells (Fig. 1H, left panel) as well as the amount of extracellular Doxil (present in the supernatant of digested and pelleted cells) (Fig. 1H, right panel) was significantly higher for c-JO4-treated spheroids, indicating better penetration and uptake of Doxil due to c-JO4-mediated epithelial junction opening. Finally, we confirmed the activity of c-JO4 in vivo, in mice with subcutaneous T84 xenograft tumors that were intravenously injected with Doxil or c-JO4 + Doxil (Fig. 1I). While all mice treated with Doxil reached the endpoint by day 50 after the start of treatment, animals treated with c-JO4 + Doxil combination survived significantly longer with 40% of mice being tumor-free at the end of the observation period (day 140). This demonstrates the therapeutic potency of c-JO4 in vivo. Notably, we have previously shown in tumor xenograft models (including ovarian cancer^11,18^, breast cancer^7,9^, and prostate cancer^9^ models) that JO4 (r-JO4) alone does not exert anti-tumor activity, i.e. tumor growth was not significantly different between groups that were injected with PBS or JO4 alone. Importantly, in all of these models, intravenous injection of JO4 one hour before the administration of chemotherapy drugs significantly enhanced chemotherapy.

Taken together, these data demonstrate that c-JO4 is functionally active.

### GLP toxicology studies in DSG2-transgenic mice

**Figure 2:**
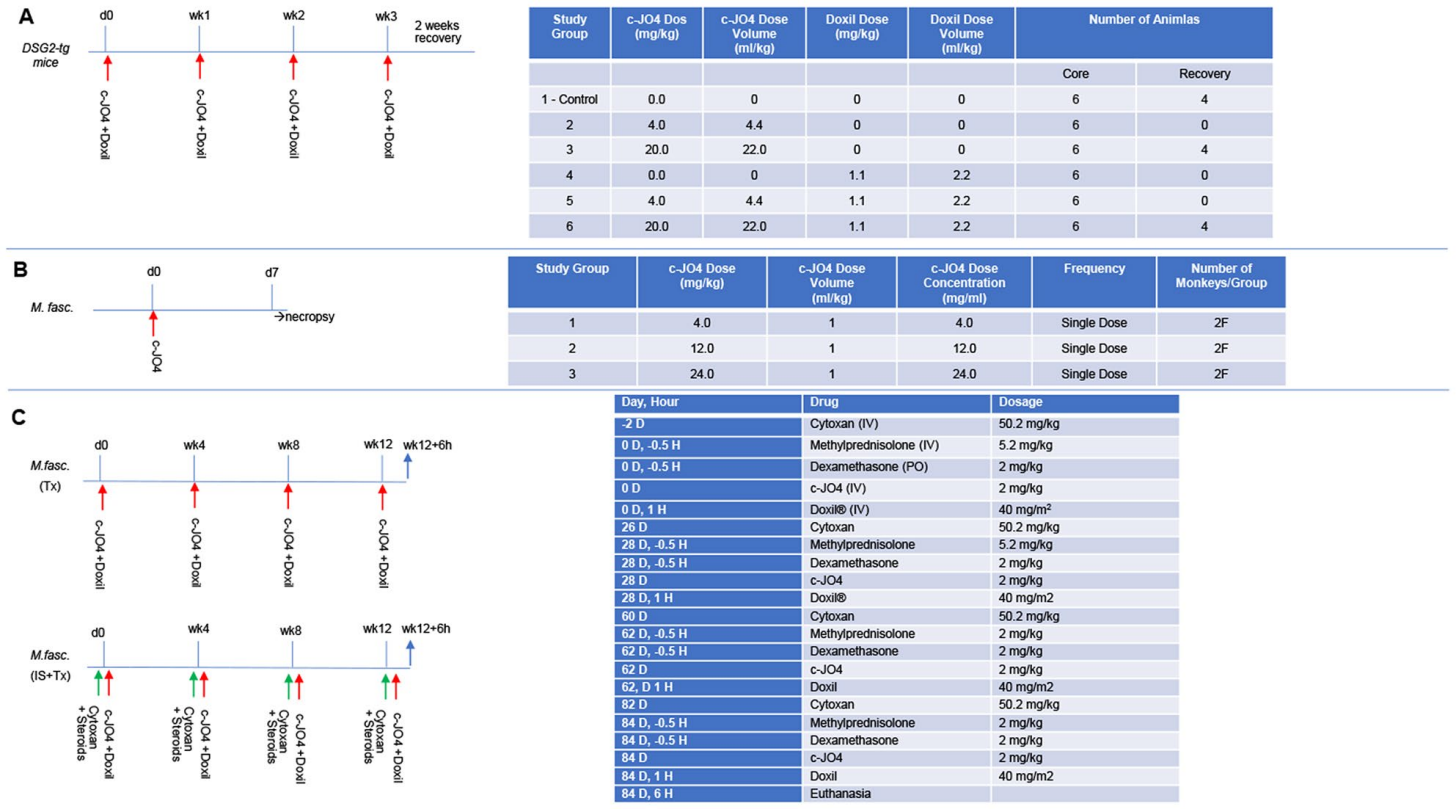
Summary of animal studies. (**A**) Four-week intravenous GLP-toxicology study of c-JO4 in female hDSG2-transgenic mice. The objectives of this study were to evaluate the potential toxicity of c-JO4 when administered intravenously together with Doxil to hDSG2-transgenic mice weekly for 4 consecutive weeks with 2 weeks of recovery. c-JO4 was injected intravenously one hour before intravenous injection of Doxil. The animals were approximately 5–11 weeks old and weighed 19.9–26.0 g at the time of the first dose administration. The study consisted of one control and five treated groups. Each group had 6 Core female mice (undergoing standard evaluations such as clinical observations, body weight, clinical pathology, organ weights and histopathology). In addition, 4 female mice per group in groups 1, 3, and 6 were designated as Recovery animals. The recovery animals were handled exactly as the Core mice but were held for at least 14 days of rest after the last dose to determine the reversibility of potential treatment-related effects. (**B**) Intravenous dose range finding study in female cynomolgus monkeys. Dose range study plan of c-JO4 in female cynomolgus monkeys. 3 groups of monkeys (N = 2) were administered with escalating doses of c-JO4 (4, 12, 24 mg/kg). Group 3 was dosed after no toxicity was observed at the previous dose level. Necropsy was performed at day 7 after administration of the c-JO4 dose to collect organ and tissue samples. (**C**) Non-GLP four-cycle toxicity /pharmacokinetics /immunogenicity study with c-JO4 + Doxil in cynomolgus monkeys. One animal received c-JO4 and, one hour later, Doxil as indicated (“Tx”). The second animal additionally received immunosuppressive drugs (“IS + Tx”). Immunosuppression consisted of Cyclophosphamide given 2 days before c-JO4/Doxil) and methylprednisolone + dexamethasone (0.5 hrs before c-JO4 injection). Timing and dosage of drug injection is shown on the right side.

These studies were performed at the GLP test site, Experimur Toxicology and Research. The objectives of this study were to evaluate the potential toxicity of c-JO4 when administered intravenously alone and in combination with Doxil to female hDSG2-transgenic mice weekly for 4 consecutive weeks with 2 weeks of recovery. The study consisted of one control and five treated groups with a 0, 4, and 20 mg/kg c-JO4 and a constant Doxil dose (1.1 mg/kg) (Fig. 2A). This dose was obtained by allometric scaling of the therapeutic dose in humans, which is 40 mg/m^2^. As in previous mouse and NHP studies with r-JO4^9,11^, c-JO4 was injected intravenously 1 h before intravenous injection of Doxil. Each group had six Core female mice (undergoing standard evaluations such as clinical observations, body weight, clinical pathology, organ weights and histopathology). In addition, four female mice per group in groups 1, 3, and 6 were designated as Recovery animals. The recovery animals were handled exactly as the Core mice but were held for at least 14 days of rest after the last dose to determine the reversibility of potential treatment-related effects. No treatment-related clinical side effects were observed for the groups that received low dose c-JO4. Treatment-related observations were limited to the mice that received 20 mg/kg of c-JO4 (Groups 3 and 6). Hypoactivity was observed for 10 of 10 mice in Group 3. Hypoactivity, cold to touch, convulsions, ptosis, and lethargy were noted for females given 20 mg/kg c-JO4 + 1.1 mg/kg Doxil (Group 6). Evaluation of group mean body weight, body weight gains and hematology revealed no test article related changes (Suppl. Fig. 1A). c-JO4 or c-JO4 + Doxil-related changes in clinical chemistry were limited to the females given 20 mg/kg JO4 (Groups 3 and 6) and included decreased serum albumin globulin, total protein, calcium, and cholesterol and increased serum A/G ratio at termination (Suppl. Fig. 1B,C). No test article-related changes in organ weight parameters were noted (data not shown).

In summary, weekly administration of c-JO4 intravenously (with and without Doxil) for 4 weeks to female hDSG2- transgenic mice caused no critical test article-related toxicity. The “No Observed Adverse Effect Level” (NOAEL) for the study was the highest dose, i.e. 20 mg/kg/injection.

### Intravenous dose range finding study in female cynomolgus monkeys

These studies were also performed in GLP compliance at Experimur Toxicology and Research. The objective of this study was to find the highest non-severely toxic dose (HNSTD) of c-JO4 when administered intravenously to cynomolgus monkeys. The study consisted of a single intravenous administration of c-JO4 with a 7-day follow up. Three c-JO4 doses (4, 12, and 24 mg/kg) were tested, and each group had 2 female monkeys (Fig. 2B). The dose range was based on the efficacy studies in xenograft tumor models^9,10,19,20^, safety studies in DSG2 transgenic mice (see above), and pilot studies in cynomolgus monkeys that were performed with 2 mg/kg r-JO4^11^.

c-JO4 was well tolerated when administered intravenously to female monkeys at dose levels up to 24 mg/kg. No treatment-related observations were noted. Evaluation of body weight, body weight gain, clinical chemistry and hematology values revealed no test article related effects. Gross necropsy observations were limited to adhesions of the cecum and rectum for one high-dose female. The adhesions appear to be a pre-existing condition and not thought to be test-article related. In histopathology analyses, mild hemorrhage and chronic inflammation were noted at the injection site for one animal given 12.0 mg/kg/day. There were no other treatment-related microscopic findings.

**Figure 3:**
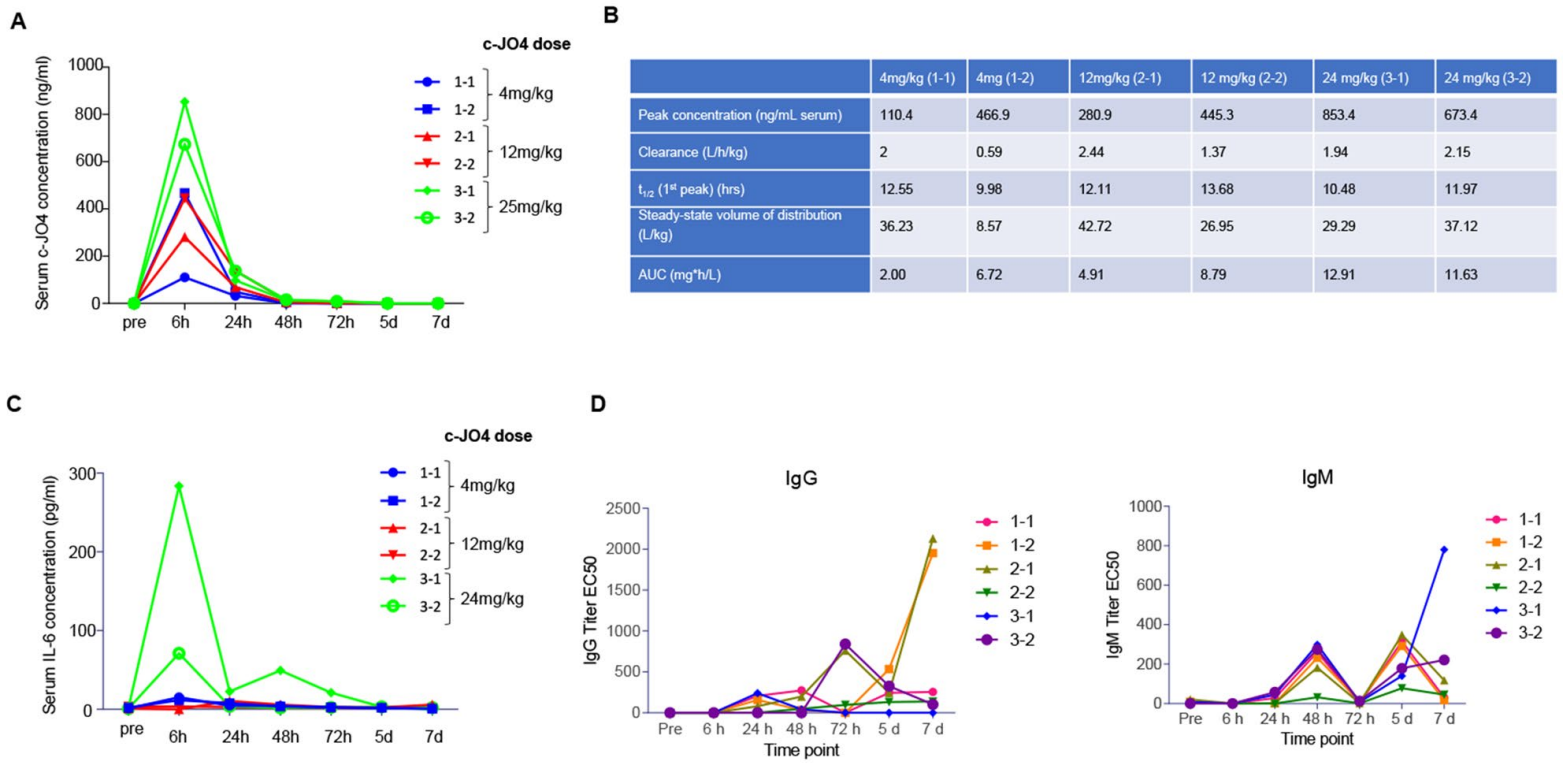
Intravenous dose range finding study in female cynomolgus monkeys. (**A**,**B**) Pharmacokinetics parameters of c-JO4 in cynomolgus monkeys. (**A**) c-JO4 concentrations in serum collected at indicated time points subsequent to c-JO4 administration were measured by ELISA. Standard curve used to quantify c-JO4 is shown on the right side. (**B**) Pharmacokinetic data calculated by using serum concentration curves of each animal. Units are as indicated. (**C**) Cytokines IL-2, IL-4, IL-5, IL-6, TNF-alpha, and IFN-gamma were measured by cytometric bead array (CBA) (NHP th1/Th2 cytokine kit from BD Biosciences). Only IL-6 was detectable. (**D**) Antibody titers present in serum against c-JO4 were measured by ELISA, using recombinant c-JO4 protein for capture and anti-NHP-IgG-HRP and anti-NHP-IgM-HRP for detection. Shown are IgM and IgG titers as IC_50_.

Blood samples were collected pre-treatment and at 6, 24, 46, 72 h, day 5 and day 7. c-JO4 Serum clearance and correlative pharmacokinetics showed a half-life of 10–12 h for all three doses (Fig. 3A,B). Animals showed a consistent clearance ranging from 1.5 to 2.5 L/h/kg for all animals except one, which had received the 4 mg/kg dose. Peak concentrations of c-JO4 in serum increased accordingly, as expected, with dosage. Serum IL-6 levels were elevated for the animals treated with 24 mg/kg with a peak at 6 h (Fig. 3C). None of the animals had pre-treatment anti-c-JO4 IgM or IgG antibodies (Fig. 3D). As expected, c-JO4 injection triggered the development of antibodies as early as at day 5 in an animal from the 4 mg/kg group and in an animal from the 12 mg/kg group.

In conclusion, no systemic treatment-related findings were noted for animals administered 4.0, 12.0 and 24.0 mg/kg/day. Injection of 24 mg/kg triggered a transient elevation of serum IL-6 not lasting more than 24 h. We used these data to determine that the HNSTD is 24 mg/kg.

### Non-GLP 4-cycle toxicity/pharmacokinetics/immunogenicity study in cynomolgus monkeys

Because c-JO4 is a viral protein, adaptive immune responses are likely to develop—particularly after repeated injection. Furthermore, Doxil contains a polyethylene glycol (PEG) coat, which, potentially can trigger anti-PEG antibody responses^21^. To assess the safety of repeated c-JO4/Doxil treatment, we designed a study in two female cynomolgus monkeys that closely reflected the treatment regimen planned in ovarian cancer patients (Fig. 2C). c-JO4 (2 mg/kg) was given intravenously one hour before IV Doxil at a dose of 40 mg/m^2^. The treatment was repeated 3 times with an interval of 4 weeks. Twelve hours after the 4th treatment cycle, animals were euthanized, and a necropsy was performed. As c-JO4 immunogenicity has already been previously noted, a common immunosuppression regimen was tested in the second animal for potential implementation in the clinical study. Immunosuppression consisted of cyclophosphamide given 2 days before c-JO4 and methylprednisolone plus dexamethasone given 30 min before c-JO4.

#### Physical health and hematological parameters

Both animals tolerated treatment well. Daily monitoring of the infusion site, food and water intake, and feces/urine did not show any abnormalities. There was no weight loss. Electrocardiograms were taken prior to each injection of c-JO4 + Doxil and results were normal (data not shown). Overall, no unexpected test article-related changes were found in hematological analyses. Immunosuppression in the “IS + Tx” animal resulted in low white blood cell/lymphocyte counts (Suppl. Fig. 2A). As expected, glucocorticoids triggered a transient dip in cortisol levels (Suppl. Fig. 2A). In both animals, c-JO4 + Doxil injection results in a small, temporary decline in C3 complement levels and red blood cell parameters (Suppl. Fig. 2B) which could be multifactorial. No treatment-related pathological or histopathological findings were listed in audited necropsy reports (see Suppl. Information).

#### c-JO4 pharmacokinetics and biodistribution

**Figure 4:**
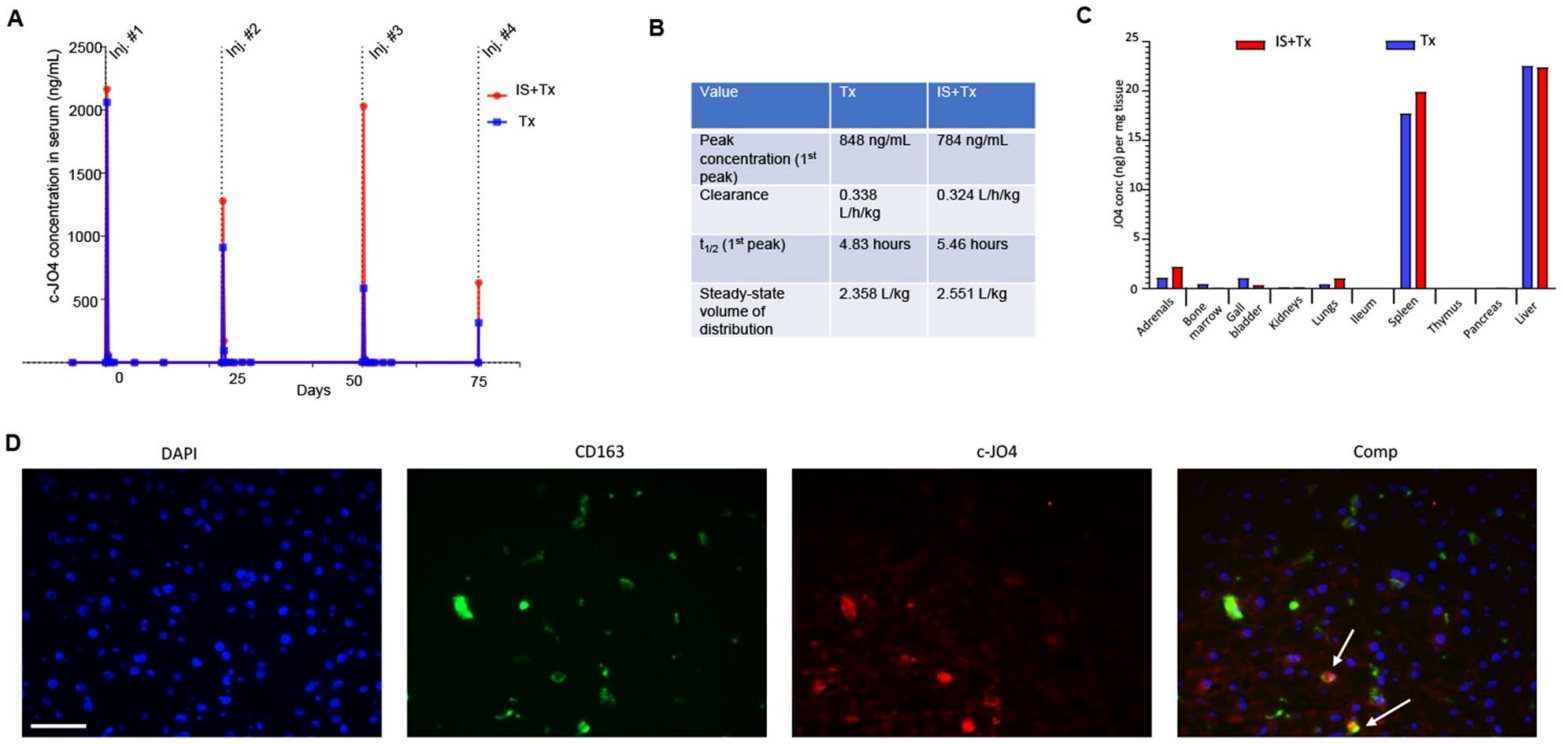
Non-GLP four-cycle toxicity /pharmacokinetics /immunogenicity study with c-JO4 + Doxil in cynomolgus monkeys. (**A,B**) c-JO4 pharmacokinetics. (**A**) c-JO4 concentration in serum collected at indicated time points as measured by ELISA. (**B**) Pharmacokinetics parameters. (**C**) c-JO4 concentration in it issues. Tissues were ground with QiaShredder and resulting tissue lysates were measured by ELISA for c-JO4. c-JO4 levels in tissue lysates were normalized for mg tissue. (**D**) Immunofluorescence with anti-CD163 and anti-cJO4 antibodies on liver sections of the “Tx” animal. Nuclei are stained blue with DAPI. The scale bar is 20 μm. In the composite panel (right) co-staining is marked by arrows.

Serum samples were taken immediately before each c-JO4 injection, and then at 3, 6, 24 h, 2, 7 and 14 days after each injection. c-JO4 concentrations were measured by ELISA (Fig.4A,B). Overall, peak c-JO4 concentrations declined from cycle 1 to cycle 4. The decline was less pronounced for the animal that received immunosuppression (“IS + Tx”). After the first cycle, c-JO4 serum half-life was 4.83 and 5.46 h for the “Tx” and “IS + Tx” animals, respectively (Fig. 4B). Six hours after the 4th c-JO4 injection, c-JO4 is mainly found in the spleen and liver (Fig. 4C). Immunofluorescence studies on liver sections showed that (83 ± 7%) of anti-c-JO4 antibody staining co-localized with staining for the macrophage/Kupffer cell marker CD163 (Fig. 4D). c-JO4 signals in the liver that do not overlap with CD163 could be derived from granulocytes, which have been shown to express low levels of DSG2 in humans^5^.

c-JO4 biodistribution was similar for both animals. c-JO4 was detected in urine at necropsy, 2 h after the last administration of c-JO4 + Doxil, at a concentration of 1.15 ng/mL and 1.37 ng/mL for the “IS + Tx” and “Tx” animals, respectively.

#### anti-c-JO4 serum antibodies

**Figure 5:**
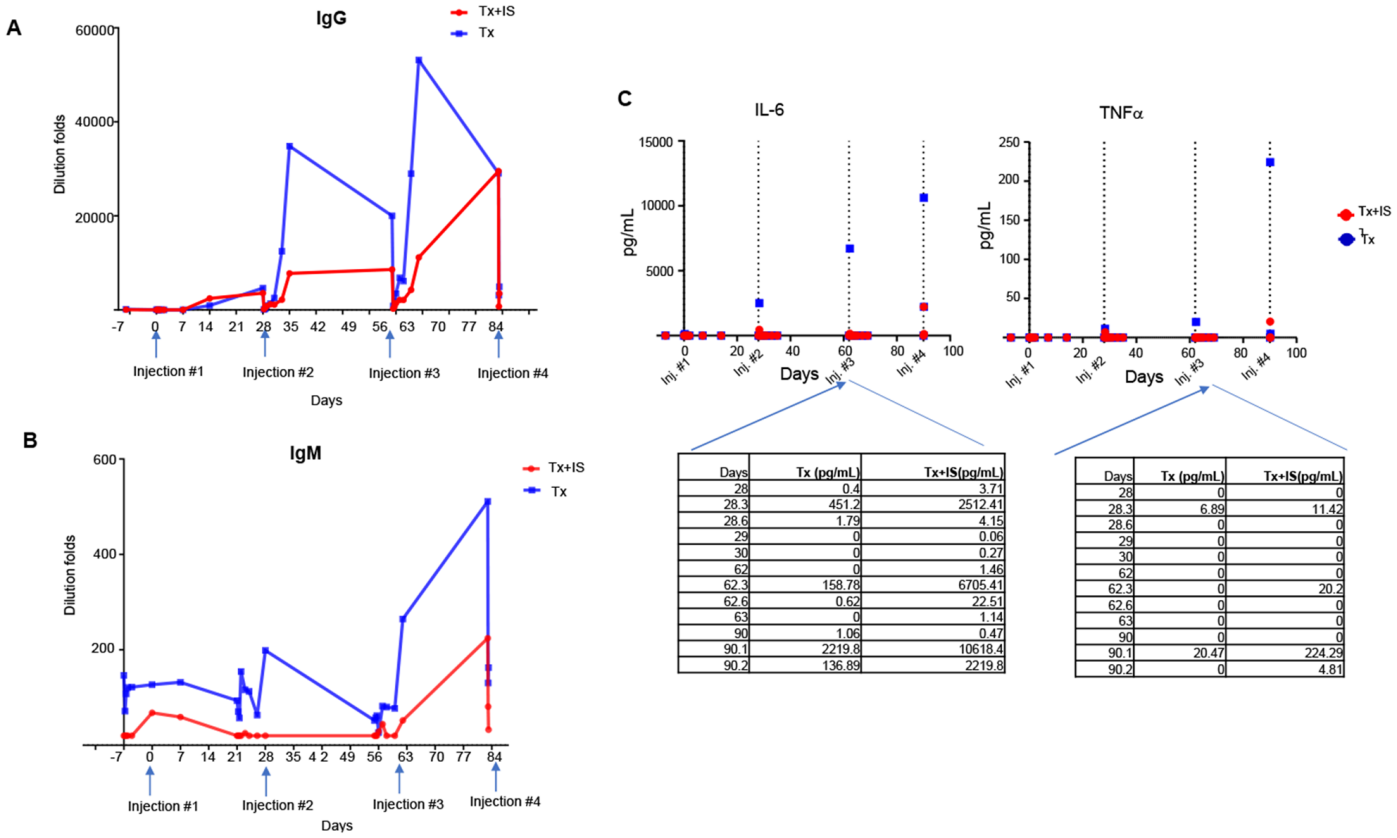
Non-GLP four-cycle toxicity/immunogenicity study with c-JO4 + Doxil in cynomolgus monkeys. (**A**,**B**) Serum anti-c-JO4 antibody titers**.** Anti-c-JO4 IgG and IgM titers were measured in at indicated time points by ELISA. (**C**) Serum cytokines. Serum samples were run on a Th1/Th2 inflammatory cytokine array using the CBA flow panel (BD Biosciences). The only cytokines above limit of detection were IL-6 and TNFα. Other cytokines tested included IL-2, IL-4, IL-10 and IFNγ. Inlet shows the values at and around each injection time point.

Serum anti-c-JO4 IgG titers increased with each cycle (Fig. 5A,B). Immunosuppression was partially effective. Intravenous injection of 2 mg/kg c-JO4 was sufficient to completely saturate anti-c-JO4 serum antibodies (Suppl. Fig. 3). We found low titers (1:20 and 1:100) of serum anti-c-JO4 IgM antibodies at day -7 in both animals (see Fig. 5B). We speculate that the animals had a simian adenovirus infection and that corresponding antibodies cross-reacted with c-JO4 to some degree.

This “priming” might explain the strong humoral response that led to the faster-than-expected clearance of c-JO4 from serum and the increasing speed of clearance with successive injections.

To study potential consequences of the transient formation of immune complexes between c-JO4 and antibodies, we measured pro-inflammatory cytokines in serum samples using an inflammatory Th1/Th2 cytokine array. The cytokines tested included interferon gamma, tumor necrosis factor (TNF), IL-6, IL-5, IL-4, and IL-2. Of these, IL-2 was not detected in any sample at any time point. Spikes in the other cytokines were detected shortly after each injection, peaking at the 2 h post-c-JO4/Doxil administration time point for each injection and increasing thereafter for subsequent injections (Fig. 5C). The highest levels were found for IL-6 and TNFα. Notable differences were seen between the two animals in IL-6 and TNF levels. In the “Tx” animal, which received no immunosuppression, IL-6 and TNFα levels reached 10,000 pg/mL and 220 pg/ml, respectively. In “IS + Tx” animal, cytokine levels were five–tenfold lower and in the non-critical range. As before the scatter in response could be due to differential exposure to related adenoviruses.

#### Doxil concentration and antibody response

**Figure 6:**
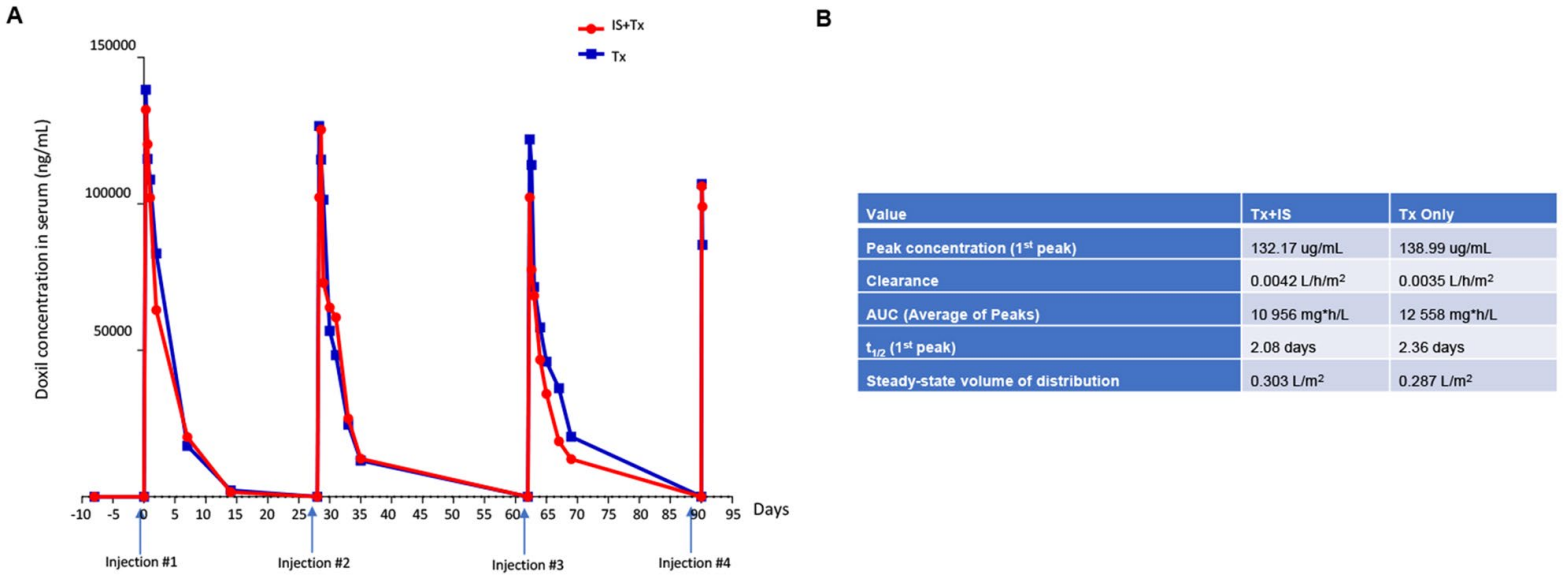
Pharmacokinetics of Doxil in cynomolgus monkeys. Doxil was detected in serum using anti-PEG antibodies and a HRP-conjugated secondary antibody. (**A**) Pharmacokinetics after each cycle. (**B**) Pharmacokinetics parameters after the first cycle.

Doxil concentration in serum was measured using an ELISA with anti-PEG monoclonal antibodies^21^ (Fig. 6A). The ELISA recognizes PEG only in particle form (e.g. Doxil). Pharmacokinetics of the Doxil in serum followed a peak within 2 h of administration, declining until about day 14 when it was no longer detectable. There were no discernible differences between the two animals in Doxil kinetics, suggesting that the immunosuppression had no significant effect on the clearance of Doxil from serum. Also, subsequent administrations did not significantly affect the pharmacokinetics of Doxil. Half-life of Doxil in the serum after the first injection was calculated to be 2.08 and 2.36 days for animal “IS + Tx” and animal “Tx”, respectively (Fig. 6B). When compared to published parameters for Doxil, clearance is a magnitude slower than in human patients (approximately 0.04 L/h/m^2^)^22^. Notably, antibody responses against PEG were not detected in serum in either animal, thus suggesting that the administration of Doxil does not trigger a humoral immune response.

#### Pre-existing anti-c-JO4 and anti-PEG antibodies in ovarian cancer patients

**Figure 7:**
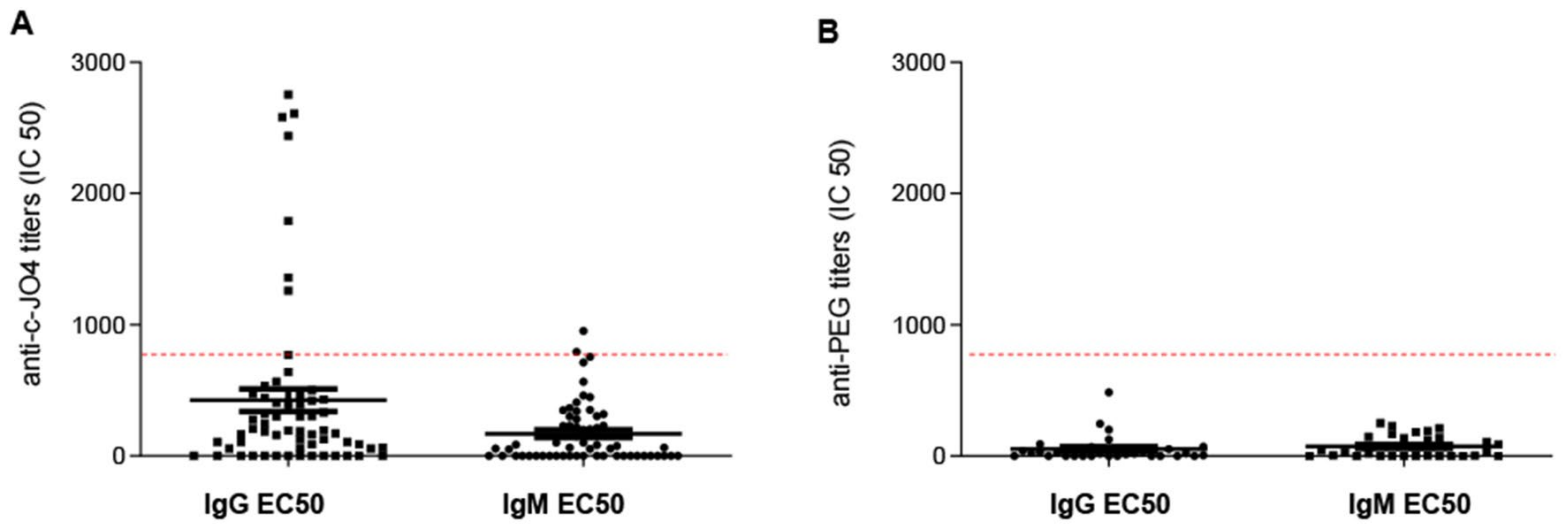
Serum antibodies against c-JO4 and PEG/Doxil in ovarian cancer patients A titer below 1:800 is not neutralizing in in vitro infection studies. Serum samples from the Fred Hutchinson Cancer Research Center was taken for analysis of antibodies against (**A**) c-JO4 (**B**) PEG/Doxil using anti-Ad3 and anti-PEG antibodies respectively in an ELISA. EC50 values are shown.

Despite the fact that approximately one third of humans have neutralizing antibodies against human adenovirus serotype 3, most of these antibodies are directed against the main capsid protein, called hexon^23^, and not the fiber protein, from which c-JO4 is derived. High-titer, pre-existing serum anti-c-JO4 antibodies could trigger anaphylactic reactions after intravenous c-JO4 protein infusion. To assess the seroprevalence of anti-c-JO4 antibodies in a relevant target population, we obtained serum samples from women with progressive, persistent or recurrent ovarian/fallopian tube cancer who have previously received standard therapies. This would be the cohort of patients considered for our clinical trial. Materials were provided by the TOR Biorepository at the Fred Hutchinson Cancer Research Center. We determined that an IC50 IgG titer of greater than 800 was neutralizing for c-JO4 in our in vitro blocking assay (Fig. 7A). With this threshold, 10% of patient samples would have neutralizing IgG anti-c-JO4 titers. These patients would have been excluded from the trial. Serum antibody titers against PEG were below the threshold in all patient samples (Fig. 7B). Clearly, an important task in the clinical trial would be to monitor the development of anti-drug antibodies along with the analysis of cytokines—and other potentially anaphylactic—responses after each treatment cycle.

## Discussion

Our goal is the clinical translation of c-JO4 in combination with Doxil for ovarian cancer therapy. A major step toward this goal was the establishment of a cGMP compliant protocol for c-JO4 manufacturing. As part of an IND application, we here performed toxicology studies with this material along with additional studies to assess immunological and biochemical parameters in animals in response to the administration of the clinical candidate protein. We also assessed and confirmed the potency of the material in a series of in vitro studies to ascertain its equivalency to previously produced r-JO4 material.

A potential concern was the safety of intravenously injected c-JO4 because the c-JO4 target receptor, DSG2, is expressed in most epithelial tissues. However, in normal epithelial tissues (that display a strict apical-basal polarization), DSG2 is trapped in lateral junctions, and thus is not readily accessible to intravenously injected ligands^5,11^. We believe that this is a compounding factor for the good c-JO4 safety profile we demonstrated here. Dose-escalation studies in both relevant animal models, DSG2 transgenic mice and cynomolgus monkey, did not trigger adverse clinical side effects in single-cycle and multi-cycle treatment regimens in combination with a clinically relevant dose of Doxil.

Intravenous c-JO4 injection is expected to trigger antibody responses which could affect the safety and efficacy of the approach. In the multi-cycle treatment study, we confirmed the development of an antibody response. This resulted in the formation of immune complexes as suggested by the transient disappearance of anti-c-JO4 IgG from the circulation immediately after c-JO4 injection (Fig. 5A). This affected the pharmacokinetics, leading to a faster clearance of c-JO4 from the blood circulation. Our immunofluorescence studies suggest that immunocomplexes containing c-JO4 were taken up by macrophages of the liver and spleen. A consequence of macrophage activation is the release of pro-inflammatory cytokines. In our study, we found elevated IL-6 and TNFα levels, and these levels increased with each treatment cycle in concordance with increased humoral immune responses. Clearly, the formation of immune complexes and downstream reactions pose a safety risk^24^. We therefore implemented a prophylactic regimen consisting of pretreatment with cyclophosphamide/Cytoxan and glucocorticoids. Cyclophosphamide has been used as a single agent before the development of platinum-based medications^25^ and was investigated as a potential co-therapeutic with paclitaxel or cisplatin in ovarian cancer patients^26,27^. Glucocorticoids mediate temporary immunosuppression, decreasing the function and/or numbers of neutrophils, lymphocytes (including both B cells and T cells), monocytes, and macrophages. Glucocorticoids are often given during chemotherapy to mitigate side effects (e.g. nausea, vomiting, and weight loss). In our studies, the combination of cyclophosphamide and glucocorticoids, given before c-JO4 injection, greatly decreased anti-c-JO4 antibody levels and blunted cytokine reactions even after the 4th cycle. In an additional upcoming GLP toxicology study, we plan to add pentostatin to the immunosuppressive regimen to further block anti-c-JO4 antibody development. Previous reports demonstrated that pentostatin plus cyclophosphamide chemotherapy safely prevented anti-immunotoxin antibody formation with uniform efficacy^28^. Immunosuppressive drugs (and Doxil) could affect potential anti-tumor immune responses, and this has to be considered if the JO4 approach will be given in combination with immune checkpoint inhibitors (used in a fraction of ovarian cancer cases of DNA mismatch repair deficiency). A potential solution to the immunogenicity of c-JO4 would be its PEGylation. PEGylated drugs (e.g. PEGylated factor VIII) are widely used clinically and have good safety profile^21^. Notably, anti-protein drug immune responses are common for immunotoxins and there is substantial clinical experience to counteract them.

Even though it was not found an issue in our NHP studies, immune responses against PEGylated drugs such as Doxil are of concern in humans. Many humans have been exposed to PEGylated medicines^21^. In a clinical trial with Doxil, up to 7% of participants developed hypersensitivity reactions^29^ and after the recent, widespread use of PEGylated liposomal mRNA COVID-19 vaccines, this percentage would probably be now even higher. The potential for emergence of anti-PEG responses will be closely monitored in our future clinical trial.

In summary, we evaluated the safety profile of c-JO4 plus Doxil in two adequate animal models under GLP-compliant conditions. We found no critical clinical side effects. The study also allowed us to determine the HNSTD for additional pre-clinical and clinical studies. Mild toxicity caused by anti-cJO4 antibody responses were observed in a multi-cycle treatment regimen. These reactions appear to be manageable as our data with glucocorticoid + cyclophosphamide pretreatment indicate. Clearly, in the clinical trial, pre-existing anti-JO4 antibodies and treatment-induced immune reactions would be monitored—especially after multiple cycles so that they can be counteracted with appropriate immune suppression.

Currently, there are no epithelial junction openers used clinically for cancer therapy. Our work could lead to a novel therapeutic approach requiring lower dosages of therapeutic agents while increasing their efficacy and tumor penetration. While we focus in this study on Doxil, successful completion of these clinical studies could have a profound impact on cancer therapy across numerous solid tumors with a number of different therapeutics—including other nanoparticle-based chemotherapy drugs, monoclonal antibodies, oncolytic viruses, and CAR T-cells.

## Methods

### Protein production

cGMP compliant (“c-JO4”) was produced by Gennova Biopharmaceuticals Ltd (Pune, India). c-JO4 is expressed in *E. coli* and accumulated intracellularly in the form of inclusion bodies (insoluble aggregates of misfolded protein lacking biological activity). c-JO4 was isolated from the cultured cells using a pressure-based cell homogenizer. Cell mass containing c-JO4 was resuspended in 10 × lysis buffer containing Tris, EDTA, Urea, NaCl, and β mercapto-ethanol (BME), pH 8.0, and subjected to cell lysis at 1000 bar. After cell disruption, the crude inclusion bodies (IBs) were washed with 1% non-ionic surfactant (Triton X100) to remove contaminating host cell impurities like DNA and proteins, followed by a water wash to remove excess surfactant and impurities. Purified IBs were recovered as settled pellet after centrifugation at 10,000×*g*. Even after multiple washing steps, IBs are mainly composed of aggregated mass of overexpressed heterologous proteins, which further needed to be solubilized in a solubilization buffer containing strong denaturants (8 M urea, Tris base, NaCl and BME, pH = 10.0) for 2 h. The pH of solubilized IBs was adjusted to 7.4 and the solubilized sample clarified by centrifugation at 15,000×*g* for 30 min followed by a 0.45 µm filtration.

Clarified and solubilized sample was subjected to Immobilized Metal Affinity Chromatography (IMAC) for further purification. IMAC was performed under denaturing conditions. This column utilizes an immobilized nickel metal chelate resin, which binds to His- tagged c-JO4 protein with a high degree of specificity. The column was equilibrated with buffer containing 8 M Urea at pH 7.4. In order to remove process- and product-related impurities, a low concentration imidazole wash was developed, and target protein eluted in purified and concentrated form using a step gradient to a buffer with a higher concentration of imidazole.

After solubilization and primary capture using IMAC, the resulting protein needed to be refolded to its native conformation: Pooled elution fractions from the capture chromatography were reduced for 30 min using 5 mM cysteine, followed by 10 × dilution in a refolding buffer containing Tris, 5% D-sorbitol, 2 M Urea, pH 8.5. Cysteine and Cystine were used as redox reagents in the refolding buffer to obtain refolded and active protein. Dimerized, refolded c-JO4 was next subjected to gel filtration chromatography to remove high and low molecular weight impurities based on their size. Phosphate buffer containing D-sorbitol was used as a working buffer for this step. The elution peak of purified product was collected and subjected to anion exchange chromatography for polishing.

The final anion exchange chromatographic step was incorporated in the process to reduce host cell proteins, residual DNA, and bacterial endotoxins. A strong anion exchange resin was packed in a glass chromatographic column. Under optimized loading and washing conditions, c-JO4 was obtained in the flow through and then buffer exchanged using Tangential Flow Filtration (TFF) to yield c-JO4 in its final buffer for infusion. The TFF system consisted of a 10 kDa Nominal Molecular Weight Cutoff cassette primed with a buffer containing PBS and 5% D-Sorbitol at pH 7.4 ± 0.2. The anion exchange chromatography flow-through fraction containing the c-JO4 was taken and concentrated to 1.0 ± 0.2 mg/ml. The concentrated protein solution was terminally filtered through a 0.2 µm sterilization grade filter. Purified c-JO4 bulk was then stored at 2 to 8 °C in a sterile PETG container. Final endotoxin levels were < 200 EU/ml.

### Therapeutics and dosing

PEGylated Liposomal Doxorubicin/Doxil was purchased from the University of Washington Medical Center, manufactured by Dr. Reddy’s Laboratories (Hyderabad, India). Methylprednisolone, dexamethasone, ceftadizime, acyclovir, cyclophosphamide (Cytoxan®), and mesna were obtained from the University of Washington Medical Center in generic forms. Ceftadizime and acyclovir are antibiotic and antiviral drugs used to protect NHPs against potential nosocomial infections during the procedure. c-JO4 was injected at the concentration indicated per animal. Doxil was dosed at 40 mg/m^2^, using the Mosteller formula: $Surface Area \left({m}^{2}\right)= \frac{\sqrt{Height \left(cm\right)*Weight (kg)}}{60}$. Drugs were diluted in physiological saline immediately prior to administration.

### Animals

#### Xenograft tumor model

Immunodeficient (CB17) mice (strain name: NOD.CB17-Prkdcscid/J) were obtained from The Jackson Laboratory (Bar Harbor, ME). Human colon cancer T84 cells (ATCC, CCL-248) were injected into the mammary fat pad (1:1 with Matrigel) of CB17 mice. c-JO4 was intravenously injected one hour before the application of PEGylated liposomal doxorubicin/Doxil. Tumor volumes were measured three times a week. Each treatment group consisted of a minimum of five mice. Mice were euthanized when tumors reached a volume of 1000 mm^3^ or displayed ulceration.

#### DSG2 transgenic mice

These mice are homozygous for the human DSG2 locus (two copies). Husbandry and genotyping are described elsewhere^17^. Mice were housed in specific-pathogen-free facilities. All mouse experiments were conducted in accordance with the institutional guidelines set forth by the University of Washington under IACUC protocol #3108–01.

#### NHP studies at experimur

Female naïve Chinese origin cynomolgus monkeys *Macaca fascicularis* (purpose-bred cynomolgus monkey) from Primate Products, Inc. (Immokalee, FL) or Envigo Global Services, Inc. were selected for use in this study. The animals were approximately 2–5 years old and weighed 2.4–4.4 kg at the time of first test article administration.

#### NHP studies at the Washington National Primate Research Center (WaNPRC)

Female *M. fascicularis* were obtained from Altasciences Inc. (Everett, WA, USA) and at the WaNPRC on the University of Washington. The studies were performed by the WaNPRC Research Support Team. c-JO4 and Doxil were filtered through 0.2 µm filters before administration into the arm vein of sedated animals. Upon completion of study, animals were sedated before euthanasia and extraction of organs for histological analysis.

### Size exclusion chromatography (SEC)

Samples were loaded onto a Superdex 200 Increase 10/300 GL column (Cytiva Life Sciences) and flowed through an Agilent 1260 series HPLC using 1 × Tris-buffered saline (TBS) at a flow rate of 1 mL/min to obtain the elution profiles.

### SDS-PAGE/western blot

Mini-Protean precast gels (Bio-Rad, Hercules, CA) with 4 to 15% gradient polyacrylamide were used. A total of 1 μg protein mixed with 2 × loading buffer (10 mM Tris–HCl, pH 6.8, 200 mM dithiothreitol [DTT], 4% SDS, 20% glycerol, 0.2% bromophenol blue) was loaded per lane. Samples were either boiled (B) for 5 min or loaded unboiled (UB). The following running buffer was used: 25 mM Tris, pH 8.3, 0.192 M glycine, 0.1% SDS. After electrophoresis, proteins were transferred to nitrocellulose and incubated with recombinant human DSG2 protein and anti-DSG2 antibodies as described previously^5^.

### Transmission electron microscopy (TEM)

Recombinant c-JO4 protein was visualized by negative-stain EM to assess its assembly status. The standard mica-carbon preparation was used with protein at 0.1 mg/ml. Sample was stained using 1% (wt/vol) sodium silicotungstate (pH 7.0) and visualized on a JEOL-1200 electron microscope at 100 kV.

### Surface plasmon resonance

Acquisitions were done on a Biacore 3000 instrument. HBS-N (GE Healthcare, Pittsburgh, PA) supplemented with 2 mM CaCl_2_ was used as the running buffer in all experiments at a flow rate of 5 μl/min. Immobilization on a CM4 sensorchip (Biacore) was performed using DSG2 at 10 μg/ml diluted in 10 mM acetate buffer, pH 4.5, injected for 10 min on an ethyl(dimethylaminopropyl) carbodiimide-N-hydroxysuccinimide (EDC-NHS)-activated flow cell. A control flow cell was activated by EDC-NHS and inactivated by ethanolamine. Different concentrations of r-JO4 and c-JO4 proteins were injected for a 3-min association time followed by a 2.5-min dissociation time, and the signal was automatically subtracted from the background of the ethanolamine-deactivated EDC-NHS flow cell. Kinetic and affinity constants were calculated using BIAeval software.

### Potency assay—Viral inhibition

A 293 cell suspension was confirmed to be > 98% viable, and their concentration adjusted to 1 × 10^5^ cells/ml, and then plated in black, flat clear bottom 96 well plates with 200 µL per well (Corning, Inc., Corning, NY). Following 18 h of incubation at 37 °C in 5% CO_2_, growth media was discarded and 62.5 µL of protein or control diluted in complete DMEM (DMEM containing 10% FBS, 1 × Pen/Strep, 1 mM Glutamax) was added to each well in quadruplicate. A total of 11 half log dilutions were tested in quadruplicate for each protein. Following 1 h incubation, 50 µL of Ad3-GFP virus (25pfu/cell) in complete DMEM were added. Two hours later, media was removed and replaced with fresh complete DMEM, and the plates were further incubated for 16–18 h at 37 °C and 5% CO_2_. The following day, GFP fluorescence was measured from the bottom read orientation at 475 nm Excitation and 505 nm Emission using a SpectraMax i3 plate reader (Molecular Devices, Inc., Sunnyvale, CA) utilizing SoftMax Pro software. Data were plotted using GraphPad Prizm (GraphPad Software, Inc., La Jolla, CA) and the IC_50_ determined using a 5-parameter non-linear fit of the sigmoidal curves using Softmax Pro software.

### Potency assay—Spheroids

T84 cells were seeded in Aggrewell 400 (Stemcell Technologies, Vancouver, Canada) at a seeding density of 200 cells per microwell (equivalent to 2.4 × 10^5^/well). Spheroids were allowed to grow in cell media (DMEM containing 10% FBS, 1 × Pen/Strep, 1 mM Glutamax) for at least 4 days to allow sufficient spheroid growth and tight junction formation. Spheroids were then collected by pipetting media through a reversible 70 µm cell strainer; trapped spheroids were flushed for further use. Approximately 1000 spheroids (1 mL) were transferred into a 24-well plate for incubation with JO4 and Doxil combinations and were incubated at 37 °C at 5% CO_2_ for the indicated time points. At the time point, spheroids were collected and dissociated with 0.05% trypsin solution (Gibco). Cells were then collected for flow cytometry measuring either Doxil fluorescence (DOX +) or mean autofluorescence intensity (MAFI) using the wavelengths 470/560 nm, corresponding to the phycoerythrin (PE) profile on a BD FACSCanto flow cytometer.

### Antibodies

Polyclonal rabbit antibodies directed against the Ad3 fiber knob and mouse monoclonal anti-Ad3 fiber knob antibodies (clone 2–1) were described earlier^17^. DSG2 antibodies were obtained from Invitrogen (Carlsbad, CA, USA). Anti-PEG antibodies were kindly provided by Dr. Steve Roffler (Academia Sinica, Taipei, Taiwan).

### Preparation of tissues for ELISA

100 mg of tissue or organ in PBS-0.05%Tween20 were homogenized using the TissueRuptor system (Qiagen), sonicated for 20 s, and subjected to three freeze–thaw cycles. Cell debris was spun down and supernatants from lysed tissues were used in the c-JO4 and Doxil ELISA at 1:5, 1:20, and 1:100 dilutions.

### Serum c-JO4 antibody ELISA

Animal blood obtained in serum separation tubes at the indicated time points were centrifuged at 5000 RPM for 10 min, after which the serum was kept at − 20 °C before use. ELISA plates were coated with c-JO4 protein (0.3 mg per well) at 4 °C overnight then blocked with 5% non-fat milk/PBS for 1 h. Serum or tissue homogenate serial dilutions (starting at 1:50, 3 × subsequent dilutions) were added for 1 h. After washing, HRP-conjugated secondary antibodies against NHP IgG or IgM (Invitrogen PA1-84631 and 62–6820 respectively) were added at 1:10,000 for 1 h. After washing, Thermo 1-Step Ultra TMB Solution (ThermoFisher Scientific) was added and the color was allowed to develop for 7 min before stopping with 2 N sulfuric acid. Absorbance readings at 450 nm were taken with a plate reader. Antibody reactivity curves were plotted with GraphPad Prism with a 4-parameter curve, and EC50 values were accordingly calculated.

### Antibodies in patients

Serum samples from ovarian cancer patients were made available from Fred Hutch Research Center (Seattle, WA). Serum samples were assessed for anti-c-JO4 antibody titers by using the protocols for c-JO4 ELISA and Doxil ELISA as outlined below. EC_50_ measures were calculated using Prism Software and plotted.

### c-JO4 ELISA

The ELISA consisted of a polyclonal rabbit antibody directed against the Ad3 fiber knob as capture antibody and a mouse monoclonal anti-Ad3 fiber knob antibody (clone 2–1) as detection antibody, using the common ELISA protocol as stated above, incorporating a standard curve using the c-JO4 protein at concentrations ranging from 100 ng/mL to 0.0064 ng/ml. The sensitivity of the ELISA was 0.5 ng/ml.

### Serum Doxil ELISA

For Doxil detection, plates were coated with anti-PEG antibodies (rAGP6 from anti-PEG, Academia Sinica, 250 ng per well) overnight at 4 °C. Plates were then blocked with 5% non-fat milk for 1 h, and serum dilutions were added after washing (starting at 1:10, 10 × subsequent dilutions) and incubated at room temperature for 1 h. This was followed up by another, different specificity anti-PEG antibody (15-2b-biotin) and incubated for 1 h. After wash, HRP-streptavidin (Jackson ImmunoResearch #016-030-084, West Grove, PA, USA) was added to wells at 0.5 microgram/mL and incubated for 1 h. Doxil standard concentration curves were generated by plotting data points and using a 4-parameter curve fit (GraphPad Prism) and were used to determine Doxil concentrations in serum. The antibodies used in the ELISA recognize PEG only in the context of particles.

### DSG2 immunofluorescence

Organs were placed in optimal cutting temperature compound (OCT, Sakura Finetek, Torrance, CA, USA) and flash frozen in liquid nitrogen. Sections were sliced at 6 mm thickness and fixed in 5% paraformaldehyde at room temperature for 15 min. Slides were then blocked and permeabilized in 5% non-fat milk in 0.05% Tween-20 PBS overnight at 4 °C. After washing, the slides were incubated with the primary antibody diluted in 2% non-fat milk/PBS for 1 h, followed by the secondary antibody diluted in 2% non-fat milk/PBS for 1 h. Slides were then mounted using VECTASHIELD Antifade Mounting Medium with DAPI (Vector Labs, Burlingame, CA, USA) before visualization using fluorescent filters under a EVOS M5000 microscope (Invitrogen). Images were overlaid using ImageJ software (NIH, Bethesda, MD, USA). The following antibodies were used on 4% para-formaldehyde-fixed OCT sections of monkey organ/tissue: anti-HAdV3 fiber knob mAb- clone 2-1; goat-α-human DSG2 (AF947, R&D Systems), mouse-α-CD163 (Biolegend #333602) with the secondaries as follows: Alexa-Fluor 594 conjugated chicken anti-rabbit IgG (Invitrogen A-21442), Alexa Fluor 488-conjugated donkey anti-goat IgG (Abcam #6881), and Alexa Fluor 488-conjugated goat anti-mouse IgG (Biolegend #405319).

### Cytokine cytometric bead array

Animal sera were taken and assessed for cytokines (Th1/Th2) using the Non-human Primate Th1/2 cytokine kit/Cytometric Bead Array (CBA) (BD Biosciences, San Jose, CA, USA). Flow data were obtained using a BD LSR II flow cytometer. Results were quantified using the BD CBA FCAP Array Software.

### Hematological analyses

Blood samples were collected into EDTA-coated tubes, and analysis was performed on a HemaVet 950FS (Drew Scientific, Waterbury, CT).

### Statistical analyses

For comparisons of multiple groups, 1- and 2-way analyses of variance (ANOVA) with Bonferroni post-hoc test for multiple comparisons were used. Statistical analysis was performed with Prism, version 6.01 (GraphPad Software Inc, La Jolla, CA). For GLP toxicology studies, continuous data were analyzed for homogeneity of variance using Levene's test. If the variances were homogeneous (p > 0.001), the data were further analyzed by one-way ANOVA. If a significant F value was observed (p ≤ 0.05), each treatment group was compared to the vehicle control group using Dunnett’s two-tailed t-test (significant at p ≤ 0.05). If Levene’s test was significant (p ≤ 0.001), an appropriate transformation was applied to the data (e.g., log-transformation or rank-transformation), and the analyses was performed on the transformed data.

### Ethical approval

The study is reported in accordance with ARRIVE guidelines. All experiments involving animals were conducted in accordance with the institutional guidelines set forth by the University of Washington. The University of Washington is an Association for the Assessment and Accreditation of Laboratory Animal Care International (AALAC)–accredited research institution and all live animal work conducted at this university is in accordance with the Office of Laboratory Animal Welfare (OLAW) Public Health Assurance (PHS) policy, USDA Animal Welfare Act and Regulations, the Guide for the Care and Use of Laboratory Animals and the University of Washington's Institutional Animal Care and Use Committee (IACUC) policies. The studies were approved by the University of Washington IACUC (Protocol No. 3108-01 for mice and 3108-05 for NHPs).

## Supplementary Information

Supplementary Information.

## Data Availability

All data can be found within the article (Figs. 1–7), Supplementary Figures (Figs.S1-S3), and Supplementary information (Pathology reports). Audited reports from the GLP test site, Experimur, are available from the corresponding author on request.
