# Supplementary material for: Translational development of a tumor junction opening technology
Source: Sci Rep. 2022 May 11;12:7753. doi: 10.1038/s41598-022-11843-z (PMC9094124; doi:10.1038/s41598-022-11843-z)
Supplement: Supplementary file 1 — Supplementary Information. [file 41598_2022_11843_MOESM1_ESM.pdf]

Supplementary information

Translational Development of a Tumor Junction Opening Technology

Jiho Kim[1,2], Chang Li[1], Hongjie Wang[1], Swarnendu Kaviraj[3], Sanjay Singh[3], Laxman Savergave[3], Arjun Raghuwanshi[3], Sucheol Gil[1], Audrey Germond[4], Audrey Baldessari[4], Bingmae Chen[5], Steve Roffler[5], Pascal Fender[6], Charles Drescher[7], Darrick Carter[1,2,8], André Lieber[1,9]

1. **Suppl. Figs. S1-S3**
2. **Pathology report for "Tx" and "IS+Tx" animals (see Fig.2C)**

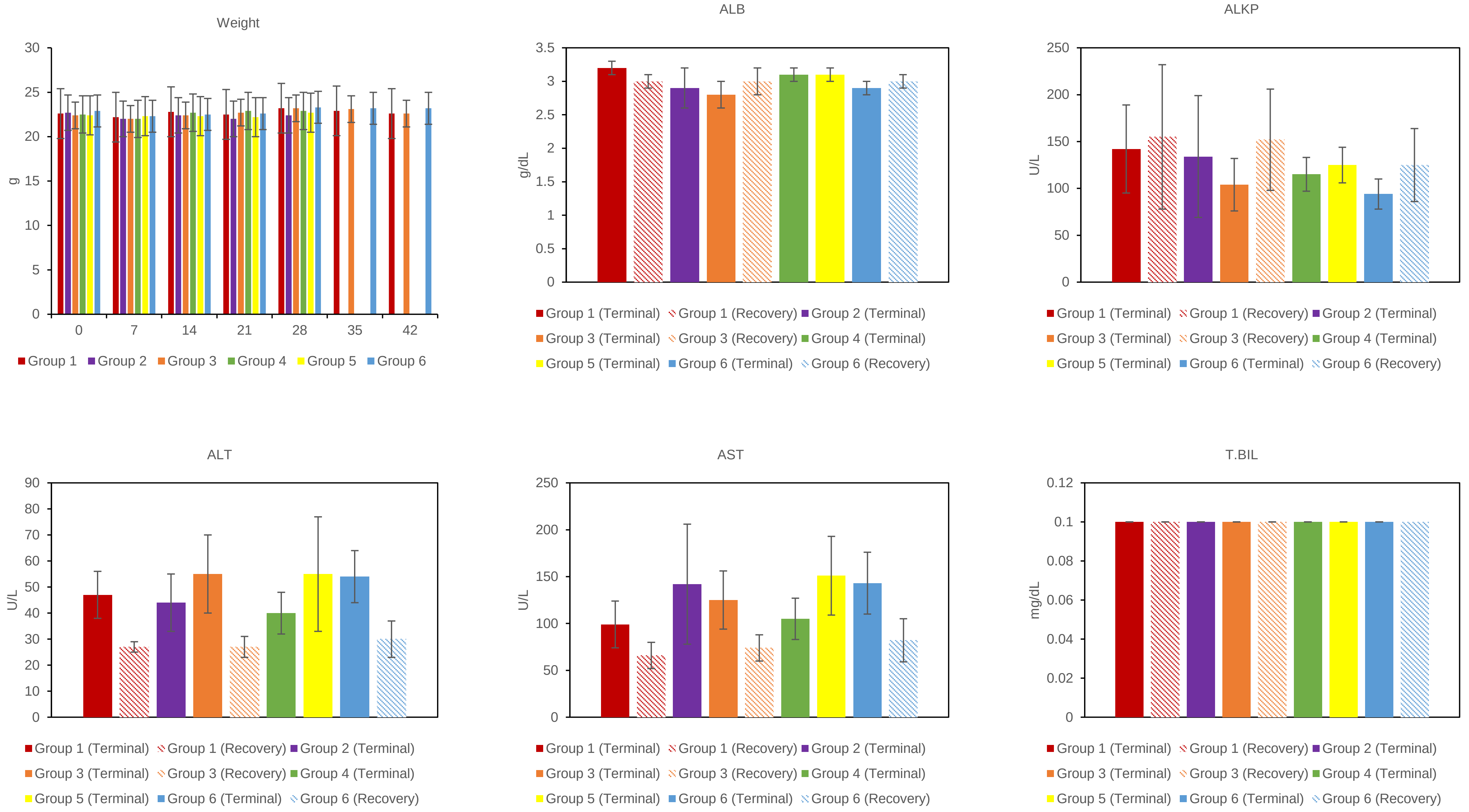

Suppl. Figure 1A. Body weight (at the indicated time points) and hematological parameters taken at the time of necropsy (“Terminal” and “Recovery” Groups) for the study in DSG2 mice shown in main Fig.2A.
Group 1: no c-JO4, no Doxil; Group 2: 4 mg/kg c-JO4, no Doxil, Group 3: 20 mg/kg c-JO4, no Doxil; Group 4: no c-JO4, Doxil; Group 5: 4 mg/kg c-J04 + Doxil; Group 6: 20 mg/kg c-JO4 + Doxil

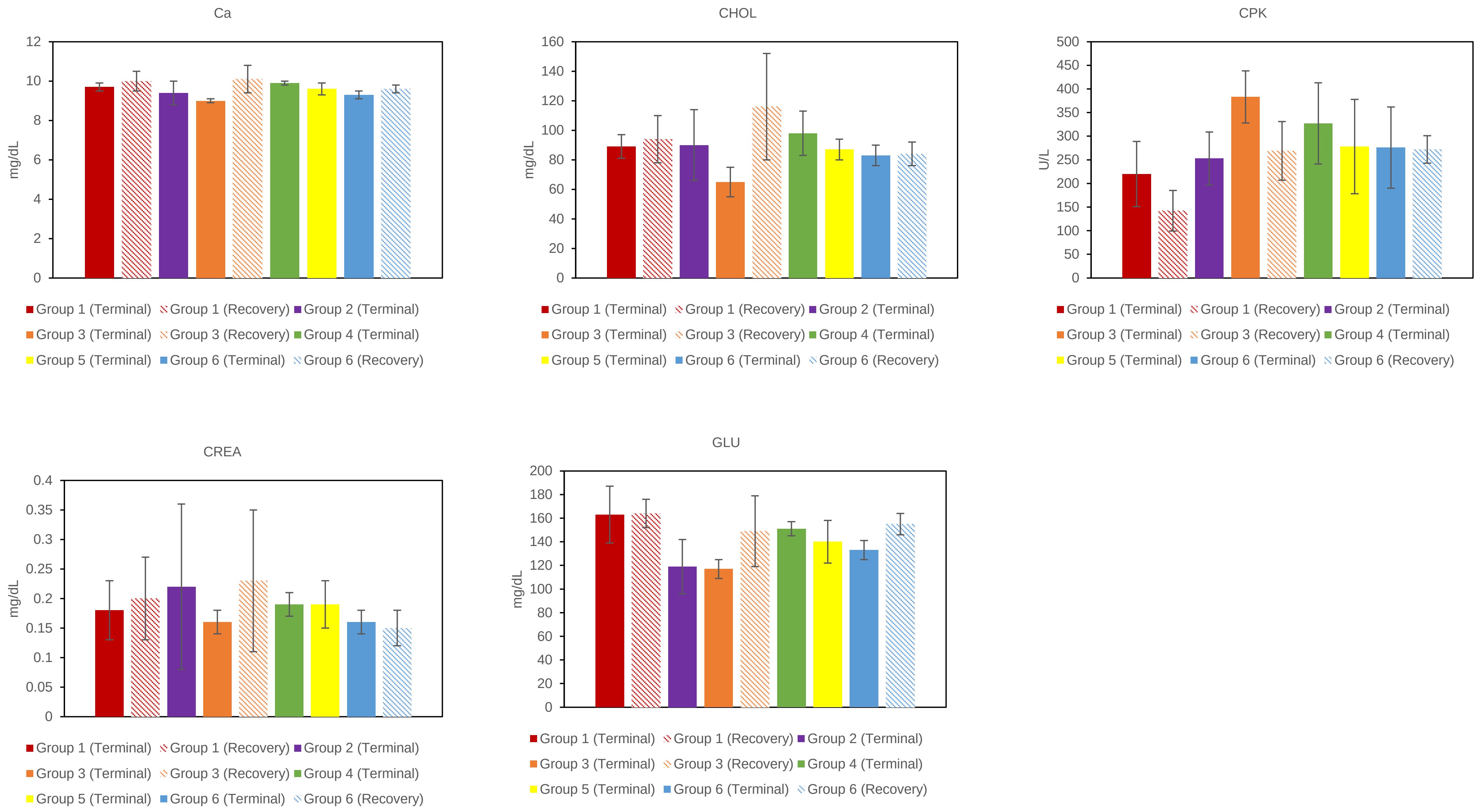

Suppl. Figure 1B. Hematological parameters taken at the time of necropsy (“Terminal” and “Recovery” Groups) for the study in DSG2 mice shown in Fig.2A.
Group 1: no c-JO4, no Doxil; Group 2: 4 mg/kg c-JO4, no Doxil, Group 3: 20 mg/kg c-JO4, no Doxil; Group 4: no c-JO4, Doxil; Group 5: 4 mg/kg c-J04 + Doxil; Group 6: 20 mg/kg c-JO4 + Doxil

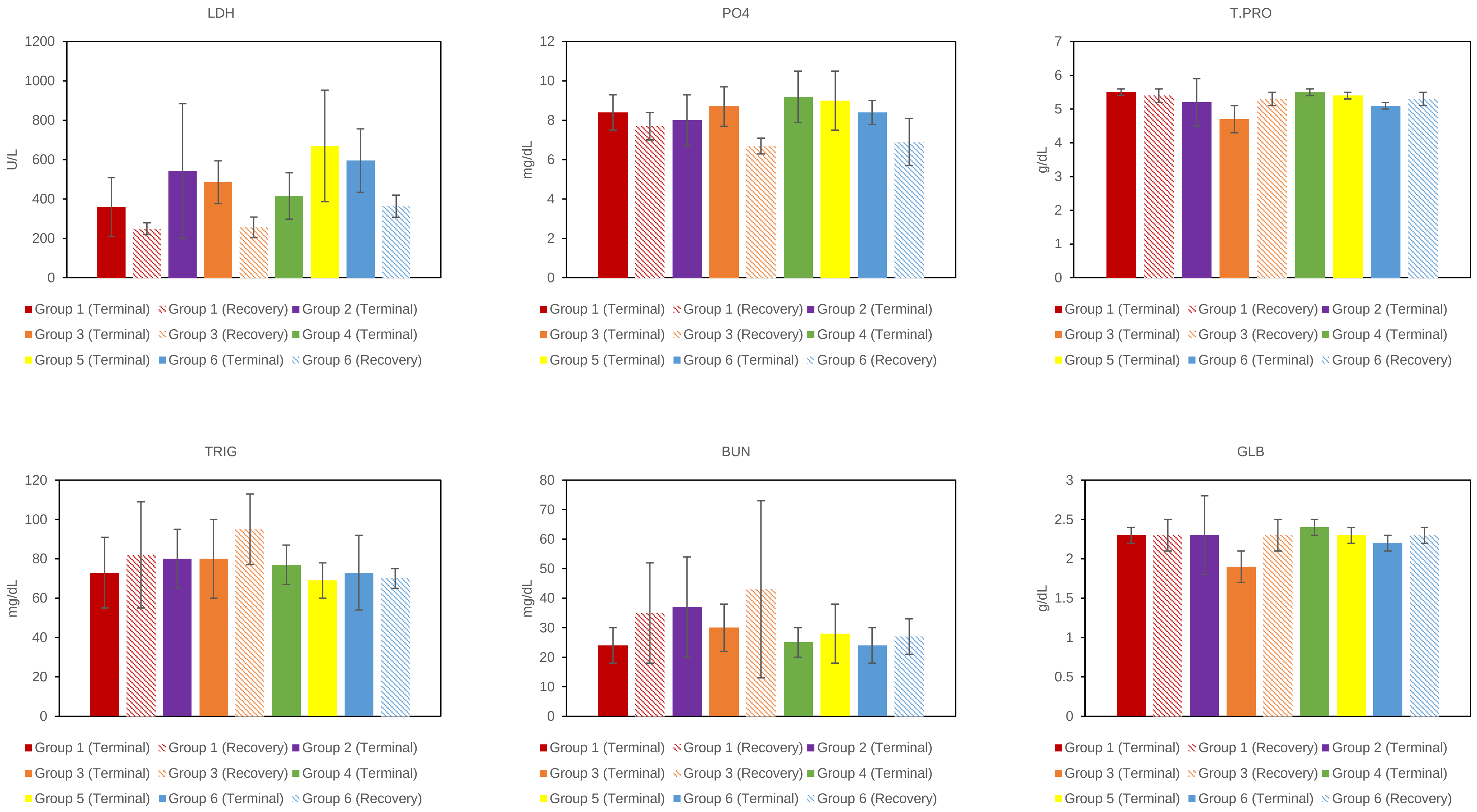

Suppl. Figure 1C. Hematological parameters taken at the time of necropsy (“Terminal” and “Recovery” Groups) for the study in DSG2 mice shown in Fig.2A.
Group 1: no c-JO4, no Doxil; Group 2: 4 mg/kg c-JO4, no Doxil, Group 3: 20 mg/kg c-JO4, no Doxil; Group 4: no c-JO4, Doxil; Group 5: 4 mg/kg c-J04 + Doxil; Group 6: 20 mg/kg c-JO4 + Doxil

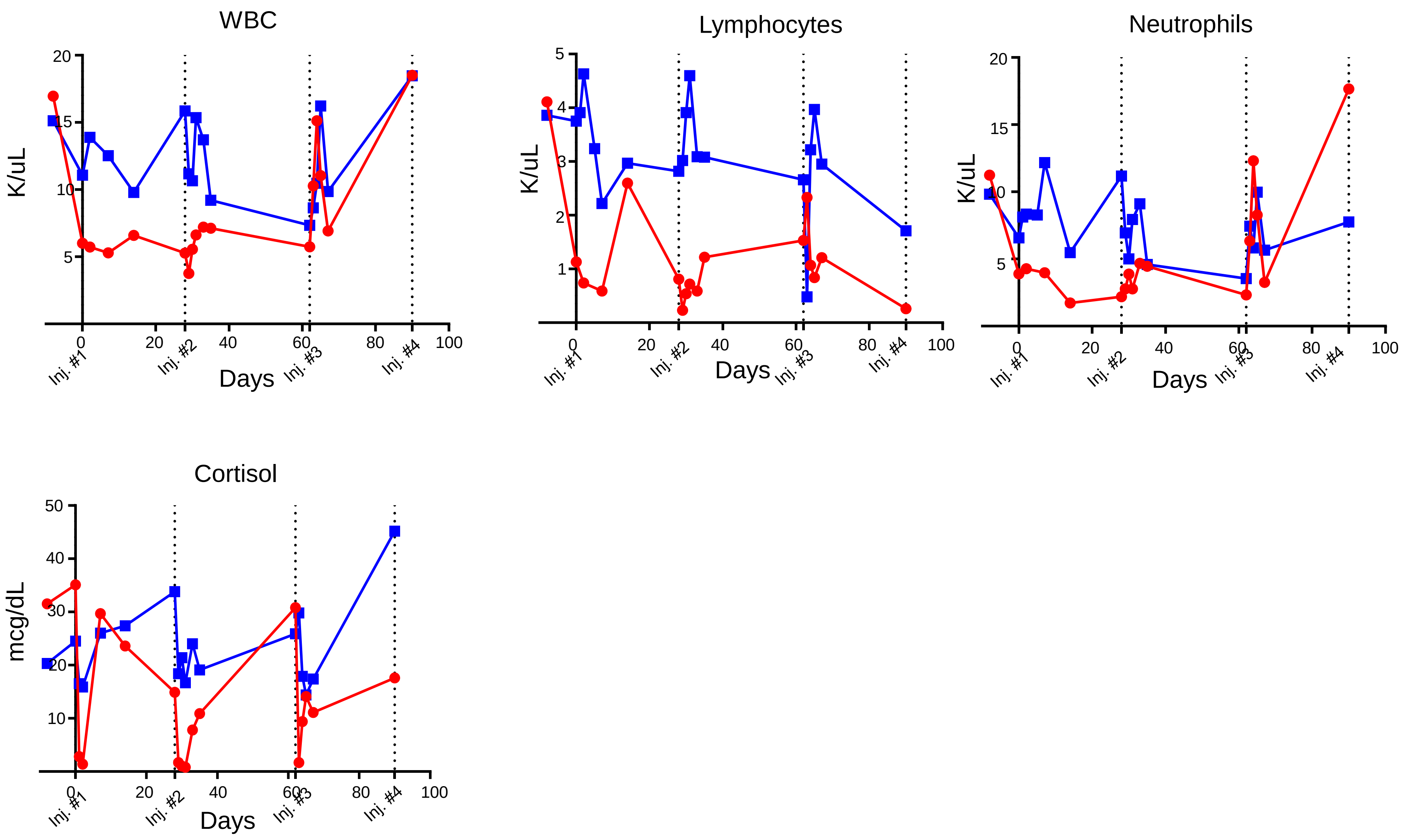

Suppl. Figure 2A. Complete blood count (CBC) and hematological parameters for the NHP study shown in Fig.2C). Blood samples were run for CBC and Hemavet testing. CBC Testing was provided by WaNPRC, while hematological parameters were read by Hemavet (Drew Scientific, Miami Lakes, FL USA).

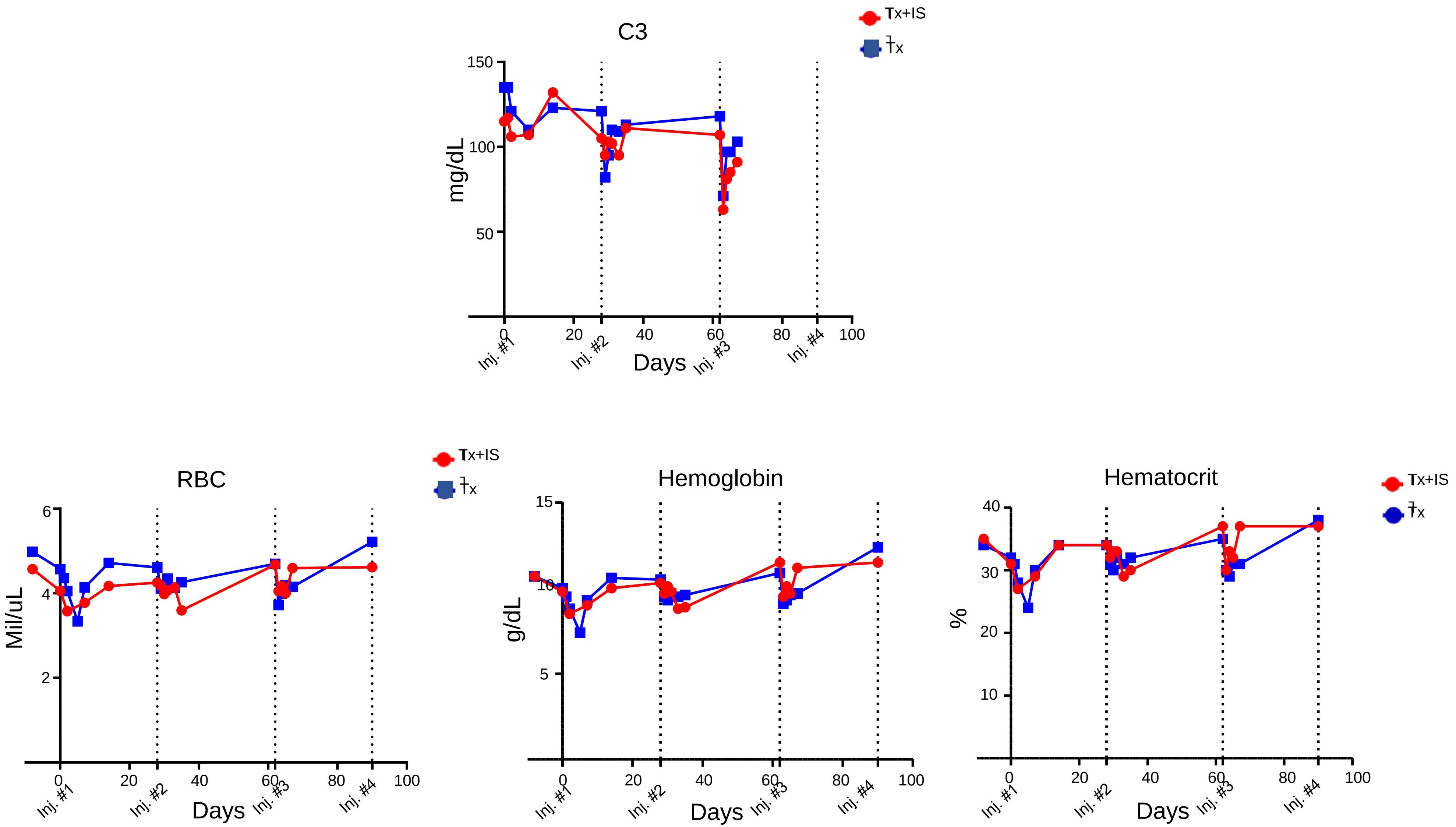

Suppl. Figure 2B. Hematological parameters for the NHP study shown in Fig.2C). Blood samples were run for CBC and Hemavet testing. CBC Testing was provided by WaNPRC, while hematological parameters were read by Hemavet (Drew Scientific, Miami Lakes, FL USA).

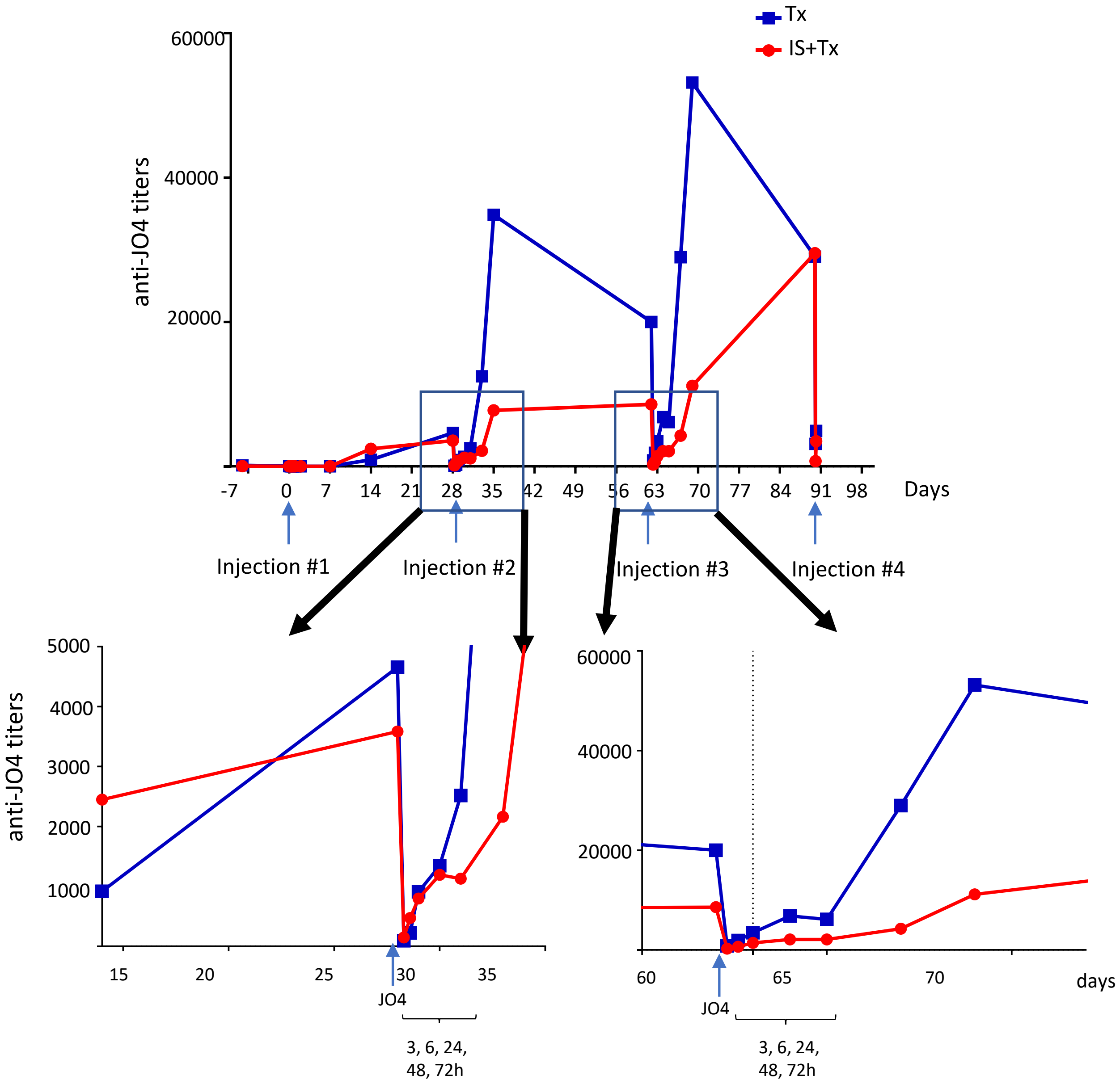

Suppl. Figure 3. Zoomed-in versions of the graphs in Fig.5A to show transient disappearance of anti-c-JO4 antibodies in serum.

Accession #___20-030_
Submission Date 5 Feb 2020

University of Washington
National Primate Research Center

DIAGNOSTIC LABORATORY BIOPSY REPORT

Requester_____AL___________ Investigator___AL_____ Animal ID #____A19198 (“IS+Tx” in Fig.2C)_____ _______

Species_______Mfl_____ Requester's Phone________________

Date of Death__5 Feb 2020_ Date of Necropsy__5 Feb 2020____Time 10am__________Pathologist _______AB_____

Nutritional Condition: ☒ Adequate ☐ Marginal ☐ Poor ☐ Obese

Other Tests Required: ☐ Sero ☐ Micro ☐ Parasit ☐ Other________________

Other Diagnostic Samples __________________

Type of report: ☒ Final 16 Oct 2020 ☐ Preliminary __5 Feb 2020_______ ☐ Amended _________

Clinical History: this animal was assigned to the “NHP study with immunosuppression” protocol. CBC and chemistry panels are unremarkable. CRP is 7.3. Fibrinogen is 498, d-dimers are 1.85.

Gross Description: 4.2 kg 3.7 year old female cynomolgous macaque in good nutritional and post mortem condition is submitted for examination after saline perfusion. There is mild dental tartar. The integuement and internal organs are unremarkable.

Gross Diagnosis(es):

Unremarkable gross examination

Gross Comments: tissues collected per research protocol.

Histological Findings:

Sections from the brain, brain stem, cerebellum, eye with optic nerve, mesenteric lymph node, submandibular lymph node, spleen, liver(rare perivascular and periportal chronic hepatitis), heart, kidneys (rare interstitial chronic nephritis and minimal fibrosis), lungs (multifocal to coalescing mild to rarely moderate chronic pneumonitis with alveolar histiocytosis and occasional congestion), pancreas, thymus (section is adipose tissue), adrenal glands, thyroid / parathyroid glands, aorta, skin, skeletal muscle, tongue, esophagus, gastrointestinal tract (multifocal mild to moderate chronic gastroenterocolitis), uterus, ovaries (focal cysts), oviducts, urinary bladder,

Final Principal Diagnosis(es):

1. Gastroenterocolitis, multifocal to diffuse, mild to moderate, chronic.
2. Pneumonitis, multifocal to coalescing, mild to moderate, chronic; lungs.
3. Chronic interstitial nephritis, multifocal, minimal; kidneys.

Histology Comments: inflammation within the gastrointestinal tract is commonly seen within the colony and etiology is likely multifactorial (nutrition, stress, parasites, etc).

Pneumonitis is overall mild to rarely moderate within these sections and no infectious agents are seen.

Nephritis is minimal and unlikely clinically significant.

Pathologist________AB________

Accession #___20-031
Submission Date 5 Feb 2020

University of Washington
National Primate Research Center

DIAGNOSTIC LABORATORY BIOPSY REPORT

Requester ________JO____ Investigator ___AL____ Animal ID # ___A19196 "Tx" in Fig.2C__
Species _____Mfl____ Requester's Phone ________________

Date of Death __5 Feb 2020 Date of Necropsy 5 Feb 2020 Time 10:30am Pathologist __AB____

Nutritional Condition: ☒ Adequate ☐ Marginal ☐ Poor ☐ Obese

Other Tests Required: ☐ Sero ☐ Micro ☐ Parasit ☐ Other________________

Other Diagnostic Samples __________________

Type of report: ☒ Final 29 Dec 2020 ☒ Preliminary 5 Feb 2020 ☐ Amended _________

Clinical History: this animal was assigned to the "NHP study with immunosuppression" protocol. CBC shows mild neutrophilia, monocytosis and lymphopenia (stress), and chemistry panels are unremarkable. Mild skin pigmentation and flakiness is noted on physical examination, possible jacket rubbing. CRP is 7, d dimers 1.99 and fibrinogen 315.

Gross Description: a 3.7 year old, 3 kg female cynomolgus macaque in good nutritional and post mortem condition is submitted after euthanasia and saline perfusion. There is minimal dental tartar and mildly flakey skin along the dorsum. Internal organs appear grossly unremarkable. Samples are collected per research protocol.

Gross Diagnosis(es):

Unremarkable gross examination

Gross Comments: tissues were collected per protocol.

Histological Findings:

Sections from the brain, brain stem, cerebellum, eye, mesenteric and axillary/inguinal lymph nodes, ovary, spleen, liver (mild to moderate centrilobular hepatocellular atrophy), heart, kidneys (multifocal, mild chronic interstitial nephritis and fibrosis), lung (multifocal, mild interstitial mixed pneumonitis, mild to moderate alveolar histiocytosis and rare partial thrombi with mixed inflammation), pancreas, thyroid glands, adrenal glands, aorta, trachea, esophagus, skin, tongue, skeletal muscle, urinary bladder, gastrointestinal tract (multifocal to diffuse, mild to moderate chronic and mildly eosinophilic gastroenterocolitis with mild submucosal lymphoid hyperplasia), and bone marrow (adequately cellular,

myeloid prodominance) with surrounding bone and skeletal muscle are examined and with exceptions of stated changes, appear unremarkable.

Uterus: a cross section of the uterus reveals a focal, less than 1 cm diameter (within this section) endometrial neoplasm, consistent with either an endometrial gland carcinoma in situ or a germ cell tumor, composed of nests and occasional acini of pleomorphic cells associated with abundant neutrophilic and macrophagic inflammation and scattered areas of necrosis. There is no evidence of mural invasion. The mass extends partially into the uterine lumen.

Final Principal Diagnosis(es):

1. Focal endometrial neoplasm, in-situ carcinoma vs germ cell.
2. Hepatocellular centrilobular atrophy, multfocal, mild to moderate; liver.
3. Pneumonitis, mild, interstitial, with alveolar histiocytosis and occasional partial thrombi with mixed inflammation; lung.
4. Mild, chronic interstitial nephritis and fibrosis; kidneys.
5. Gastroenterocolitis, multifocal to diffuse, mild to moderate with mild lymphoid hyperplasia.

Histology Comments: the uterine mass is an incidental finding, and does not appear invasive however the cell morphology supports an in-situ carcinoma or germ cell neoplasm. Immunohistochemical stains would be necessary to differentiate. This is unlikely associated with the experimental protocol but may be contributing to the skin pigment changes seen clinically. The other findings are commonly seen background changes (liver, kidney and gastrointestinal changes) or possibly associated with catherization (pulmonary partial thrombi and inflammation).

Pathologist______AB__________
